# Psychological Resources in Adults with Cancer: A Systematic Review of Quantitative Studies

**DOI:** 10.3390/cancers18152525

**Published:** 2026-08-06

**Authors:** Morgiane Bridou, Léonore Robieux

**Affiliations:** 1 Psychopathology and Processes of Change Laboratory, Distance Education Institute (IED), Paris 8 University, 2 Rue de la Liberté, 93526 Saint-Denis, France; 2 Parisian Social Psychology Laboratory, Distance Education Institute (IED), Paris 8 University, 2 Rue de la Liberté, 93526 Saint-Denis, France

**Keywords:** psycho-oncology, psychological resources, cancer survivorship, quality of life outcomes, psychological distress, positive health, systematic review

## Abstract

Background/Objectives: Patients facing cancer’s challenges develop various coping strategies. This systematic review identifies the psychological resources that influence their mental health and quality of life. Methods: Following PRISMA guidelines, we searched Medline, PsycINFO, PubMed, Science Direct, Springerlink, and Wiley for empirical studies published between 2010 and 2025 for articles containing the term “cancer” and one or more terms relating to psychological resources. Including studies focused on peer-reviewed articles evaluating at least one psychological resource and one mental health outcome in adult cancer patients. A total of 95 studies (*N* = 21,683) were included after a formal screening process. Results: Risk of Bias in Non-randomized Studies—of Exposures (ROBINS-E) assessments of the risk of bias showed the overall quality of the included studies, with 89% of studies having a low or moderate risk of bias. The review identified 16 psychological resources. We described each resource’s impact based on the strength of current empirical evidence. Optimism, resilience, hope, and self-compassion emerged as the most prominent predictors of reduced psychological distress. By analyzing study designs, these resources were organized into a functional hierarchy reflecting their specific roles within the coping process. Conclusions: Based on this categorization, we propose a conceptual model illustrating the interplay between these resources. This framework suggests a theoretical basis for future empirical validation and provides directions for designing targeted psychological interventions.

## Simple Summary

Patients diagnosed with cancer face significant challenges, leading them to develop various coping mechanisms. This research aims to identify and categorize the specific psychological resources—such as personality traits, self-evaluations, and adaptive behaviors—that help patients maintain their mental health and quality of life during treatment. By reviewing 95 studies involving over 21,000 participants, the authors sought to determine which psychological factors are most effective in supporting patient well-being. The findings led to the creation of a new theoretical model that explains how different types of resources function throughout the coping process. This research provides the scientific community with a clearer understanding of the psychological drivers behind patient resilience, offering a valuable foundation for developing more effective, personalized psychological support and interventions in oncology.

## 1. Introduction

Cancer is a major global public health concern because of its high prevalence, substantial individual and societal burden, and considerable economic impact [1]. In 2022, the International Agency for Research on Cancer reported nearly 20 million new cancer cases and 9.7 million cancer-related deaths worldwide and projected a 77% increase in cancer incidence by 2050 [1]. According to these latest estimates, the most common types of cancer are breast, lung and colorectal cancers. Looking beyond statistics and pathophysiological data, cancer survivors face huge challenges in every area of their lives, especially in the family, marital, and social spheres [2]. These challenges often have negative emotional repercussions (e.g., anxiety, sadness, anger, feelings of guilt, frustration, helplessness, inferiority, and loss of hope) that can lead to psychopathological problems (e.g., anxiety disorders, depression, psychotraumatic disorders) [3] that profoundly affect the quality of life and psychosocial functioning of cancer patients and their families [2].

Given the seriousness of these impacts and the associated risk of relapse or death, adapting to cancer is essential for improving patients’ experiences and subjective well-being [4]. Hence, cancer patients’ subjective experiences have been studied intensively, with the aim of developing new therapeutic approaches for maintaining and improving their quality of life and well-being [5]. This work has enabled researchers to identify and characterize the psychological resources underlying the mobilization, adoption, or strengthening of salutogenic proactive strategies for coping with cancer.

For many decades, clinical psychology tended to adhere to a deficit model of human functioning and focused on detecting, understanding, and attenuating suffering and mental health disorders, including for cancer patients [6]. However, a paradigm shift occurred in 2000, when Martin Seligman and Mihaly Csikszentmihalyi [6] introduced the concept of positive psychology, which encourages researchers to investigate not only individuals’ vulnerabilities, but also the strengths and resources that contribute to their happiness and fulfillment [7]. Personality traits and psychological attributes have since been shown to play key roles in improving personal satisfaction and fulfillment [8].

Psychological resources are conceptualized as endogenous protective factors—internal predispositions that empower individuals to mobilize the competencies required for adaptive coping in the face of profound life stressors [9]. From a fundamental perspective, these resources are defined by their functional utility as latent assets when activated [10]. Hobfoll’s Conservation of Resources (COR) theory defines these resources not as static personality traits, but as dynamic assets that individuals must proactively invest and protect [11]. While personality traits remain relatively stable over time, psychological resources are inherently more malleable [12]. Consequently, psychological resources represent the internal reservoir of assets available to the individual, whereas coping refers to the functional mobilization of these specific assets in response to situational challenges [11].

In line with the positive psychology paradigm, the last two decades have seen extensive research on how various psychological resources impact the physical and mental health of cancer patients, the results of which complement the abundant literature on psychological distress factors [13,14]. This research has highlighted the positive impacts of many psychological resources on patients’ well-being and the role these resources play in helping patients adopt coping behaviors, which manifest in various ways [15]. The multifactorial and transactional model of health provides a comprehensive framework for understanding how dispositional resources, psychosocial determinants, and transactional factors—such as illness representations and coping—interact to influence psychological and physical health outcomes [16]. By integrating these components, the model clarifies how internal assets function either as vulnerabilities or as protective resources that guide the coping process throughout the illness.

In a field that is increasingly focusing on protection factors and well-being, the current article describes a systematic review of quantitative, peer-reviewed studies on the impacts of psychological resources on cancer patients’ mental health. While individual constructs have been studied, a significant research gap persists regarding the lack of conceptual distinction between internal assets and their behavioral mobilization. This review addresses this ambiguity by explicitly isolating psychological resources from coping strategies. Furthermore, unlike existing literature focused on isolated variables, this study provides an integrative mapping of these resources through a comparative evidence-based framework. Our aim was to summarize current knowledge on this issue, ascertain the main resources that have been studied, indicate their role in the case of cancer, and identify possible research gaps to open the way for new research and therapeutic applications. To this end, we formulated the following research questions:

(1) Which psychological resources impact cancer patients’ mental health outcomes and quality of life?

(2) What role do psychological resources play in helping patients cope with cancer?

## 2. Materials and Methods

The present review of research on cancer patients’ psychological resources was designed, conducted, and reported in accordance with the PRISMA guidelines for systematic literature reviews [17]. The review protocol was prospectively registered in the PROSPERO database (No.: CRD420251021051). The completed PRISMA 2020 checklist is provided as Supplementary Material to this manuscript.

### 2.1. Information Sources

We conducted the literature search on 1 October 2025, searching the following electronic databases: Medline, PsycINFO, PubMed, Sciencedirect, SpringerLink, and Wiley.

### 2.2. Search Strategy

We searched for articles published between 2010 and 2025, using as search terms the names of the 72 psychological resources described in the literature (e.g., autonomy, gratitude, hope, humor, resilience, self-determination, self-efficacy, spirituality) and 13 words referring to cancer (e.g., cancer, tumor, carcinoma, sarcoma). This 15-year window was selected because the field of positive psychology and psychological resource research in oncology has experienced exponential growth and a major paradigm shift during this period. Article titles or abstracts had to include the name of at least one psychological resource and one cancer term to be included in the initial sample. A full list of search terms is available as Supplementary Material (Table S1). Our choice of search terms was guided by Peter et al.’s [18] systematic review of psychological resources in spinal cord injury and Robieux and Bridou’s [4] study of psychological resources in chronic somatic diseases. We devised a specific draft search strategy for each database. For example, the draft search strategy for PsycINFO was AB,TI(cancer* OR tumor* OR oncolog* OR carcinoma OR malignan* OR neoplasm* OR metasta* OR sarcoma* OR leukemia OR lymphoma OR mesothelioma OR myeloma OR myelodysplastic) AND AB,TI(Altruis* OR compassion OR Empathy OR forgiveness OR generosity OR Gratitude OR Helpfulness OR “Interpersonal ability*” OR “Interpersonal skill*” OR Kindness OR Niceness OR “Social abilit*” OR “social competence*”). A list of all the draft search strategies is available as Supplementary Material (Table S2). In line with our eligibility criteria, the search strategies included terms only in English and French.

### 2.3. Eligibility Criteria

Language: We included only studies published in English or in French.

Population: We included only studies whose participants were adults (age ≥ 18 years) with active cancer (all sites, stages I to IV) who were undergoing treatment (surgery, chemotherapy, radiotherapy, etc.). We excluded studies whose participants were cancer patients in remission or candidates for screening, but we placed no restrictions in terms of medical or psychiatric comorbidities.

Intervention: We included quantitative studies, whether transversal (comparative or not, with or without a control group) or longitudinal (comparative or not, with or without a control group), that began and ended while participants were undergoing treatment. Several study designs were excluded to ensure the conceptual and statistical homogeneity of the corpus. Qualitative studies were excluded because their idiographic approach and diverse definitions of psychological constructs do not allow for a standardized comparison of the statistical relationships between resources and health outcomes. Interventional studies were also excluded to avoid confounding effects; since interventions intentionally modify the natural mobilization of resources, they would obscure the observation of ‘spontaneous’ resource dynamics during cancer treatment. Finally, grey literature (e.g., dissertations, book chapters) and non-peer-reviewed articles were excluded to maintain a high threshold of methodological rigor and ensure that all included evidence had undergone stringent external validation.

Outcomes: We included all psychological resources and the following mental health outcomes: anxiety, depression, psychological distress, PTSD, stress, quality of life, and wellbeing. We excluded other outcomes, such as specific health beliefs (e.g., health locus of control), physical or physiological measures (e.g., treatment side effects, pain scales without psychological components), or behavioral compliance (e.g., adherence to chemotherapy or medication).

### 2.4. Selection Process

We used Covidence systematic review software (Veritas Health Innovation, Melbourne, Australia; available at www.covidence.org) to help with every stage of the article selection process, for which we adopted a double screening methodology, as this type of approach may be more reliable than single screening [19]. As a first stage, both researchers independently evaluated the titles and abstracts of all the articles included in the initial sample. Inter-rater reliability was “substantial” (Cohen’s Kappa = 0.63). Disagreements were resolved by consensus. Both researchers then independently evaluated the full texts of the articles retained at the end of the first stage. Disagreements on whether to include or exclude an article were resolved by discussion.

The search was strictly limited to the electronic databases mentioned above; no additional manual searches were conducted. We used Covidence systematic review software to facilitate every stage of the article selection process, including the automatic identification and removal of duplicate records. For the screening stages, a double-blind methodology was adopted. While automation tools handled duplicates, no automated classifiers or AI-assisted tools were used to evaluate study eligibility; all titles, abstracts, and full texts were screened manually by both researchers independently to ensure maximum accuracy.

### 2.5. Data Collection

Each researcher independently collected data from half the included studies and checked the data the other researcher had collected from the other half of the studies. Again, disagreements on whether to include or exclude data were resolved by discussion.

Data items: The variables extracted were psychological resources (see Search Strategy) and anxiety, depression, quality of life, and well-being. We accepted all types of valid psychometric measures used to evaluate these variables, and we did not place any restrictions on the number of time points at which outcomes were measured. Supplementary Materials (Tables S3 and S4) list all the evaluation instruments used in the studies to assess, respectively, psychological resources and outcomes.

### 2.6. Risk of Bias Assessment

We assessed the risk of bias in the included studies using the Risk of Bias in Non-randomized Studies of Exposures (ROBINS-E) tool [20]. ROBINS-E provides a dedicated methodology for observational research by systematically evaluating bias across key domains: confounding, selection of participants, classification of exposures, departures from intended exposures, missing data, measurement of outcomes, and selection of the reported results. Each study was categorized as having a low, some concerns, high, and very high risk of bias. The integration of this state-of-the-art tool allowed for a robust, finely tuned, and transparent appraisal of evidence quality, significantly strengthening the methodological validity of our systematic review. Supplementary Material (Table S6) shows the risk of bias for each of the included studies.

## 3. Results

**Figure 1:**
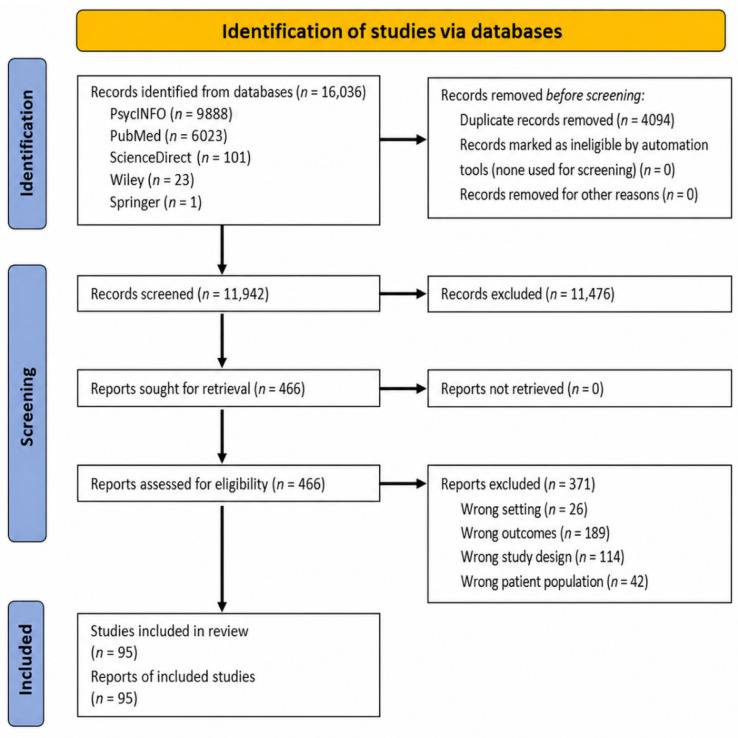
Flowchart of the systematic literature review.

The literature search yielded 16,036 results, 4094 of which were duplicates and therefore removed automatically. Screening the titles and abstracts of the remaining 11,942 articles gave us an initial sample of 466 articles, whose texts we reviewed in full. This second screening stage led us to eliminate a further 371 articles that did not meet our eligibility criteria in terms of their outcomes (notably health-specific outcomes, e.g., treatment adherence, or physical symptom management) (*n* = 189), their design (*n* = 114), their study population (*n* = 42), or their setting (*n* = 26). Thus, at the end of the two-stage screening process, we included 95 articles in our review (see Figure 1). In terms of design, 72 (75.5%) of these studies were transversal, 21 (22.4%) were longitudinal, and 2 (2.1%) had a control group. Regarding geographical distribution, the included studies were balanced across two main regions: West (*n* = 47, 49%) and Asia (*n* = 46, 48%), with two studies using a mixed sample (2%).

### 3.1. Participants

The studies involved a total of 21,683 cancer patients, with sample sizes ranging from 17 to 1085 participants (mean = 230, SD = 177); a comprehensive overview of the methodological and clinical characteristics of each included study is provided in Supplementary Material (Table S5). Participants’ ages ranged from 18 to 100 years, and 63.6% (13,916) of participants were women. The studies included all types and sites of cancer, with the most common being breast cancer and digestive system cancers (see Table 1), and all four cancer stages (I to IV). Almost half the studies (44/95 = 46.3%) included participants covering all four stages, and only 16 studies (16.8%) included just participants with nonmetastatic cancers. Participants were either outpatients (56 studies = 59.0%), inpatients (16 studies = 16.8%), a mixture of inpatients and outpatients (16 studies = 16.8%), community-based (4 studies = 4.2%), or not specified (3 studies = 3.2%).

### 3.2. Psychological Resources

The included studies evaluated a total of 16 psychological resources, with each study evaluating at least 3 different resources. This list represents an exhaustive extraction of all psychological resources documented in the fully reviewed cohort of studies. Resilience was the most frequently studied resource (25 studies), followed by post-traumatic growth (15 studies), hope (15 studies), optimism (15 studies) and sense of coherence (12 studies). Fewer studies examined spirituality (6 studies), mindfulness (6 studies), self-compassion (5 studies), positive affect (4 studies), self-efficacy (4 studies), self-esteem (4 studies), and benefit-finding (5 studies). The least commonly studied resources were creativity, self-acceptance, gratitude, and meaning in life (1 study each).

To identify each psychological resource’s role and function in coping with cancer, we classified them into three theoretical categories, in line with Bruchon-Schweitzer and Boujut’s [16] integrative model of how individuals cope with threatening and adverse situations: (1) dispositional resources, which directly impact well-being; (2) evaluative resources, which impact an individual’s ability to evaluate a situation and themselves; and (3) resources used as coping strategies. This classification was based on how each resource was conceptualized and tested by the authors of the included studies (i.e., according to their research hypotheses, statistical models—such as mediation or moderation—and interpretation of results). In cases where a resource was measured as a primary health outcome rather than a predictor or mediator, it was labeled as an ‘Issue’.

The most frequently studied health outcomes were depression (44 studies) and anxiety (35 studies), followed by quality of life (28 studies). Other outcomes were studied less frequently, with 12 studies mentioning psychological distress, 6 studies mentioning well-being, and only 2 studies mentioning post-traumatic stress.

Table 2, Table 3, Table 4, Table 5, Table 6, Table 7, Table 8, Table 9, Table 10, Table 11, Table 12, Table 13, Table 14, Table 15, Table 16 and Table 17 summarize the roles of each psychological resource and its association with health outcomes. The ‘Role’ column is populated only for studies that explicitly integrated the resource into a transactional or causal model.

#### 3.2.1. Resilience

Resilience is the ability to live, succeed, and grow in the face of adversity, uncertainty, conflict, failure, and stressful situations [21,22]. It involves a dynamic learning process: Positive emotions strengthen resilience, which enables a return to homeostasis, which in turn enables the development of resistance [23,24]. As shown in Table 2, resilience was tested against seven outcomes across 33 analyses from 24 studies. The pattern was consistent: 19 of 24 associations with adverse mental health outcomes were protective (16 significantly), and all nine associations with quality of life and well-being were positive and significant. This effect remained consistent across five different analytical methods, suggesting the finding is not dependent on a specific statistical choice. The only association not supported by evidence was insomnia, based on a single non-significant test (β = 0.17, *ns* [25]). Of the 24 studies that examined resilience, 19 (76%) considered it to be dispositional, one evaluative, one adaptive, one considered it an outcome of the coping process, and three did not define a specific role. Resilience has a consistent evidence base for clinical integration as a dispositional resource.

**Table 2:** Associations of resilience and health outcomes in cancer survivors.

Role	Associated Variable	Type of Analysis	Coefficient	Study ID
	*Mental Health*			
Dispo.	Anxiety	SEM	β = −0.20 **	[26]
Dispo.	Anxiety	Regr.	β = −0.38 ***	[27]
Dispo.	Anxiety	Regr.	β = −0.11	[28]
Issue	Anxiety	Regr.	β = 1.98 (unstandardized)	[29]
Eval.	Anxiety	Regr.	β = −0.24 **	[30]
Dispo.	Anxiety	*t*-tests	t = 5.12 ***	[31]
Cop.	Anxiety	Regr.	β = −0.27 **	[32]
Dispo.	Anxiety	Regr.	β = −0.13	[33]
Dispo.	Depression	MANOVA	F = 3.16 *	[34]
Dispo.	Depression	Regr.	β = −0.47 ***	[27]
Dispo.	Depression	Regr.	β = −0.16 *	[35]
Issue	Depression	Regr.	β = −19.79 ** (unstandardized)	[29]
Eval.	Depression	Regr.	β = −0.25 **	[30]
-	Depression	Corr.	r = −0.54 ***	[36]
-	Depression	Corr.	r = −0.49 ***	[37]
-	Depression	*t*-tests	t = 3.1 *	[31]
Cop.	Depression	Regr.	β = −0.03	[32]
Dispo.	Depression	Regr.	β = −0.27 ***	[28]
Dispo.	Depression	Regr.	β = −0.17 *	[33]
Dispo.	Insomnia	Regr.	β = 0.17	[25]
Dispo.	Psychological distress	Regr.	β = −0.01 ***	[38]
Dispo.	Psychological distress	Regr.	β = −0.24 ***	[39]
Dispo.	Psychological distress	Regr.	β = −0.75 *	[40]
Dispo.	Psychological distress	SEM	β = −0.59 **	[41]
	*Well-being and perceived health*			
Dispo.	Quality of life	Regr.	β = 0.14 *	[35]
Dispo.	Quality of Life	Regr.	β = 0.61 ***	[42]
Dispo.	Quality of life	ANOVA	F = 42.86 ***	[43]
Dispo.	Quality of life	ANOVA	F = 3.70 *	[44]
Dispo.	Quality of life	Regr.	r^2^ = 0.28 *	[27]
Dispo.	Quality of life	Regr.	β = 0.26 ***	[45]
Dispo.	Quality of life	SEM	β = 0.55 ***	[46]
Dispo.	Quality of life	Regr.	β = 0.17 *	[47]
Dispo.	Well-being	Regr.	β = 0.22 *	[31]

Legend. This table summarizes the findings from the systematic review regarding resilience. It details the theoretical role attributed to the resource (Dispo. = Dispositional; Eval. = Evaluative; Cop. = Coping), the associated mental health or quality of life variables, the type of statistical analysis (SEM = Structural Equation Modeling; Regr. = Regression; Corr. = Correlation; *t*-tests = Student’s *t*-tests; ANOVA = Analysis of Variance), and the observed coefficients. Coefficient marked “unstandardized” is raw regression coefficients (B) rather than standardized β, per the source study; its sign and significance remain interpretable, but its magnitude should not be compared directly to standardized estimates elsewhere in this table. Significance levels: * *p* < 0.05, ** *p* < 0.01, *** *p* < 0.001.

#### 3.2.2. Post-Traumatic Growth

Post-traumatic growth refers to positive cognitive and behavioral changes following a traumatic or threatening event. Such changes may include forming new representations of resources, becoming more accepting of one’s limits and vulnerabilities, and developing better interpersonal relations [48]. In contrast to resilience and optimism, which describe how individuals adapt successfully to adversity, post-traumatic growth applies to people who are transformed by their fight against adversity [49]. Table 3 reports 25 associations from 16 studies. The pattern is genuinely inconsistent rather than merely heterogeneous in metric: significant associations with both anxiety and depression were found in both directions across studies (e.g., anxiety: r = −0.37 ** [50] and r = −0.26 * [41] vs. r = 0.13 ** [51]; depression: r = −0.37 ** [50] and r = −0.26 ** [41] vs. r = 0.17 ** [51] and r = 0.38 ** [52]), with remaining tests non-significant. Three of four associations with quality of life and basic truth were positive and significant. Interpretation is complicated by the direction modeled. Of the nine studies using inferential analyses, five (62.5%) treated post-traumatic growth as an outcome of coping rather than as a resource acting on mental health [53,54,55,56,57]. Only four studies modeled post-traumatic growth as a predictor with a specified role (dispositional [50,58] or coping [59,60]), and even these were mixed, while [58] and [59] found null direct effects on quality of life and depression, respectively. Given the largely inconsistent findings, post-traumatic growth’s evidence requires further research before any claim of clinical utility.

**Table 3:** Associations of Post-Traumatic Growth and health outcomes in cancer survivors.

Role	Associated Variable	Type of Analysis	Coefficient	Study ID
	*Mental Health*			
-	Anxiety	Corr.	r = 0.13 **	[51]
Issue	Anxiety	Regr.	β = 0.59 ***	[53]
Issue	Anxiety	Regr.	β = 0.89	[54]
-	Anxiety	Corr.	r = −0.26 *	[41]
-	Anxiety	Corr.	r = −0.37 **	[50]
-	Anxiety	Corr.	r = −0.18	[52]
-	Anxiety	Corr.	r = 0.08	[26]
Issue	Anxiety	Regr.	β = −0.18	[56]
Cop.	Anxiety	Regr	β = −0.13*	[60]
-	Depression	Corr.	r = 0.17 **	[51]
Issue	Depression	Regr.	β = −0.64 ***	[53]
-	Depression	Corr.	r = 0.00	[61]
Cop.	Depression	SEM	β = −0.24	[59]
Issue	Depression	Regr.	β = −3.53	[54]
-	Depression	Corr.	r = −0.26 **	[41]
-	Depression	Corr.	r = −0.37 **	[50]
-	Depression	Corr.	r = 0.38 **	[52]
Cop.	Depression	Regr	β = −0.33***	[60]
Issue	Psychological distress	Regr.	β = −0.15	[57]
-	PTSD	Corr.	r = 0.28 ***	[62]
-	PTSD	Corr.	r = 0.12	[52]
	*Well-being and perceived health*			
-	Basic truth	Corr.	r = 0.83 *	[63]
Dispo.	Quality of life	Regr.	β = −0.01	[58]
Dispo.	Quality of life	Regr.	β = 5.48 ** (unstandardized)	[50]
Issue	Quality of life	GLM	SE = 0.15 ***	[55]

Legend. This table summarizes the findings from the systematic review regarding post-traumatic growth. It details the theoretical role attributed to the resource (Issue = Issue; Cop. = Coping; Dispo. = Dispositional), the associated mental health or quality of life variables, the type of statistical analysis (Regr. = Regression; Corr. = Correlation; SEM = Structural Equation Modeling; GLM = General Linear Model), and the observed coefficients. Coefficient marked “unstandardized” is raw regression coefficients (B) rather than standardized β, per the source study; its sign and significance remain interpretable, but its magnitude should not be compared directly to standardized estimates elsewhere in this table. Significance levels: * *p* < 0.05, ** *p* < 0.01, *** *p* < 0.001.

#### 3.2.3. Hope

Rand and Cheavens [64] define hope as “the perceived ability to produce pathways to achieve desired goals and to motivate oneself to use those pathways.” Hope has a positive effect on the mental health of cancer patients in that it reduces stress [65], depression, and anxiety [28,32,66,67,68,69,70,71,72,73,74], and improves quality of life [47,75]. Table 4 reports 22 associations from 15 studies. The pattern is fully consistent in direction: all associations with anxiety and depression are negative, and both associations with quality of life are positive, with 20 of the 22 tests statistically significant. The only non-significant findings—both for the same study [76] (anxiety and depression)—do not contradict the overall direction but indicate this study alone provides weaker support for the association. Of the 15 studies that examined hope, 10 (66.67%) considered it to be a dispositional resource in the transactional process of coping with cancer [28,32,66,67,68,69,70,71,72,73,74], two studies (13.3%) considered it to be an adaptive dimension [32,65], and two studies (13.3%) considered it to arise from the transactional process of coping with cancer [70,75]. The remaining study (6.67%) did not define a specific role [68]. The direction and significance of associations did not vary according to the role attributed to hope by the authors. Hope is one of the strongest and most consistent evidence bases for clinical integration as a dispositional resource.

**Table 4:** Associations of hope and health outcomes in cancer survivors.

Role	Associated Variable	Type of Analysis	Coefficient	Study ID
	*Mental Health*			
Dispo.	Anxiety	Regr.	β = −0.36 ***	[71]
Dispo.	Anxiety	Regr.	β = −0.22 **	[74]
Dispo.	Anxiety	Regr.	β = −0.22 *	[69]
Dispo.	Anxiety	Regr.	β = −0.09	[76]
Dispo.	Anxiety	Regr.	β = −0.45 ***	[73]
Dispo.	Anxiety	Regr.	β = −0.31 **	[66]
Cop.	Anxiety	Regr.	β = −0.27 **	[32]
-	Anxiety	Corr.	r = −0.62 ***	[68]
Dispo.	Depression	Regr.	β = −0.39 ***	[71]
Dispo.	Depression	Regr.	β = −0.42 ***	[67]
Issue	Depression	Regr.	β = −0.43 **	[70]
Dispo.	Depression	Regr.	β = −0.54 *	[69]
Dispo.	Depression	Regr.	β = −0.08	[76]
Dispo.	Depression	Regr.	β = −0.30 ***	[73]
Dispo.	Depression	Regr.	β = −0.32 **	[66]
Cop.	Depression	Regr.	β = −0.35 **	[32]
Dispo.	Depression	Regr.	β = −0.18 **	[28]
-	Depression	Corr.	r = −0.65 ***	[68]
Dispo.	Depression	Regr.	β = −0.41 ***	[72]
Cop.	Stress	Regr.	β = −0.32 **	[65]
	*Well-being and perceived health*			
Issue	Quality of Life	Regr.	β = 0.33 *	[75]
Dispo.	Quality of life	Regr.	β = 0.18 *	[47]

Legend. This table summarizes the findings from the systematic review regarding hope. It details the theoretical role attributed to the resource (Dispo. = Dispositional; Cop. = Coping; or Issue = Issue), the associated mental health or quality of life variables, the type of statistical analysis (Regr. = Regression; Corr. = Correlation), and the observed coefficients. Significance levels: * *p* < 0.05, ** *p* < 0.01, *** *p* < 0.001.

#### 3.2.4. Optimism

Optimism involves having generally positive expectations concerning future events, viewing the world positively, being inclined to feel positive emotions, and being prepared to take action because one expects positive outcomes [77]. Table 5 reports 21 associations, all from inferential analyses. The pattern is fully consistent: all 16 associations with adverse mental-health outcomes (anxiety, depression, psychological distress) are negative, 14 of which are statistically significant—the two non-significant estimates (depression, path β = −0.15 [78]; distress, β = −0.20 [79]) still point in the protective direction. All associations with quality of life and well-being are positive and significant. The negative coefficient reported for [80] reflects functional impairment (lower is better) rather than a positive-valence outcome and should be read in the same protective direction as the other quality-of-life findings. All the studies that examined optimism assessed it via inferential analyses and considered it to be a dispositional resource in the transactional process of coping with cancer [33,47,66,73,76,78,79,80,81,82,83,84,85,86,87]. Optimism is one of the strongest and most consistent evidence bases for clinical integration as a dispositional resource.

**Table 5:** Associations of optimism and health outcomes in cancer survivors.

Role	Associated Variable	Type of Analysis	Coefficient	Study ID
	*Mental Health*			
Dispo.	Anxiety	Regr.	β = −0.26 ***	[76]
Dispo.	Anxiety	Regr.	β = −0.19 *	[73]
Dispo.	Anxiety	Regr.	β = −0.25 **	[66]
Dispo.	Anxiety	Regr.	β = −0.22 **	[84]
Dispo.	Anxiety	Regr.	β = −0.39 **	[33]
Dispo.	Anxiety	Regr.	β = −0.93 **	[78]
Dispo.	Anxiety	Regr.	β = −0.51 **	[81]
Dispo.	Depression	Regr.	β = −0.30 ***	[76]
Dispo.	Depression	Regr.	β = −0.19 **	[73]
Dispo.	Depression	Regr.	β = −0.25 **	[66]
Dispo.	Depression	Regr.	β = −0.02 **	[85]
Dispo.	Depression	Regr.	β = −0.04 **	[82]
Dispo.	Depression	Regr.	β = −0.51 *	[81]
Dispo.	Depression	Regr.	β = −0.48 **	[33]
Dispo.	Depression	Path	β = −0.15	[78]
Dispo.	Psychological distress	Regr.	β = −0.20	[79]
	*Well-being and perceived health*			
-	Quality of life	Corr.	r = 0.31 **	[87]
Dispo.	Quality of life	Regr.	β = 0.22 ***	[86]
Dispo.	Quality of Life	SEM	β = −0.62 ** (reversed score)	[80]
Dispo.	Quality of life	Regr.	β = 0.17 *	[47]
Dispo.	Well-Being	Regr.	β = 0.19 *	[83]

Legend. This table summarizes the findings from the systematic review regarding optimism. It details the theoretical role attributed to the resource (Dispo. = Dispositional), the associated mental health or quality of life variables, the type of statistical analysis (Regr. = Regression; Path = Path Analysis; Corr. = Correlation; SEM = Structural Equation Modeling), and the observed coefficients. Significance levels: * *p* < 0.05, ** *p* < 0.01, *** *p* < 0.001.

#### 3.2.5. Sense of Coherence

Sense of coherence is a stable psychological disposition that indicates a person’s level of confidence within their environment. This confidence is based on perceiving the world as understandable (events are structured and predictable), manageable (the resources needed to overcome difficulties are available), and meaningful (challenges are worth rising to) [88]. Table 6 reports 14 quantified associations from eight studies (one additional study, Ref. [89], reported a quantitative parallel trend between sense of coherence and quality of life without a statistical association coefficient, and is discussed narratively rather than included in the quantitative synthesis). For mental health, the pattern is fully consistent: all six associations with anxiety and depression are negative. For quality of life, seven of eight estimates are positive, both correlational [90,91] and regression-based [92,93,94,95,96] designs; only one study [97] (β = −0.18 *) diverges, reporting a negative association with quality of life. The coefficients reported for [92] (depression, β = −1.94 ***; quality of life, β = 5.07 ***) are unstandardized regression coefficients rather than standardized β. Seven of the nine studies (77.78%) that examined sense of coherence via inferential analyses considered it to be a dispositional resource in the transactional process of coping with cancer [78,92,93,94,96,97,98], one study (11.11%) considered it to be an evaluative resource [95], and one study (11.11%) considered it to be a coping resource [99]. As with the other resources reviewed, the theoretical role attributed did not affect the direction of the association found. Overall, the evidence for sense of coherence is established and consistent across both mental-health and quality-of-life outcomes—though the single divergent finding for quality of life [97] warrants replication before firm conclusions are drawn for that specific outcome.

**Table 6:** Associations of sense of coherence and health outcomes in cancer survivors.

Role	Associated Variable	Type of Analysis	Coefficient	Study ID
	*Mental Health*			
Dispo.	Anxiety	Regr.	β = −0.32 ***	[98]
Dispo.	Anxiety	Regr.	β = −0.43	[78]
Dispo.	Depression	Regr.	β = −1.94 *** (unstandardized)	[92]
Cop.	Depression	SEM	β = −0.51 ***	[99]
Dispo.	Depression	Regr.	β = −0.52 ***	[98]
Dispo.	Depression	Regr.	β = −0.19 *	[78]
	*Well-being and perceived health*			
Dispo.	Quality of life	Regr.	β = −0.18 *	[97]
-	Quality of life	Corr.	r = 0.44 ***	[90]
Eval.	Quality of life	Regr.	β = 0.34 ***	[95]
Dispo.	Quality of life	Regr.	β = 5.07 *** (unstandardized)	[92]
-	Quality of life	Corr.	+	[89]
-	Quality of Life	Corr.	r = 0.29 **	[91]
Dispo.	Quality of life	Regr.	β = 0.04 **	[96]
Dispo.	Quality of Life	Regr.	β = 0.30 **	[93]
Dispo.	Quality of life	Regr.	β = 0.50 ***	[94]

Legend. This table summarizes the findings from the systematic review regarding sense of coherence. It details the theoretical role attributed to the resource (Dispo. = Dispositional; Eval. = Evaluative; Cop. = Coping), the associated mental health or quality of life variables, the type of statistical analysis (Regr. = Regression; SEM = Structural Equation Modeling; Corr. = Correlation), and the observed coefficients. Coefficients marked “unstandardized” are raw regression coefficients (B) rather than standardized β, per the source study; their sign and significance remain interpretable, but their magnitude should not be compared directly to standardized estimates elsewhere in this table. ‘+’ reported a quantitative parallel trend between sense of coherence and quality of life but did not report a statistical association coefficient. Significance levels: * *p* < 0.05, ** *p* < 0.01, *** *p* < 0.001.

#### 3.2.6. Spirituality

Spirituality is the ability to give meaning to one’s life and to feel connected to the present moment: to oneself, to others, to nature, and to the divine [100]. Table 7 reports seven associations from four studies. The pattern is largely consistent: all five associations with anxiety and depression are negative and significant. The only exception is psychological distress ([57], r = 0.16, *ns*), which points in the opposite direction but does not reach significance and should not be read as contradicting the overall pattern. Both associations with quality of life are positive and significant. All four studies that examined spirituality via inferential analyses considered it to be a coping resource in the transactional process in cancer [72,101,102,103]. Spirituality shows a consistent but not sufficiently extensive evidence base, supporting further research before its systematic clinical integration.

**Table 7:** Associations of spirituality and health outcomes in cancer survivors.

Role	Associated Variable	Type of Analysis	Coefficient	Study ID
	*Mental Health*			
-	Anxiety	Corr.	r = −0.53 *	[104]
Cop.	Depression	Regr.	β = −0.76 ***	[101]
Cop.	Depression	Regr.	β = −0.18 ***	[72]
-	Depression	Corr.	r = −0.62 **	[104]
-	Psychological distress	Corr.	r = 0.16	[57]
	*Well-being and perceived health*			
Cop.	Quality of Life	Regr.	β = 0.72 ***	[103]
Cop.	Quality of Life	Regr.	β = 0.28 ***	[102]

Legend. This table summarizes the findings from the systematic review regarding spirituality. It details the theoretical role attributed to the resource (Cop. = Coping), the associated mental health or quality of life variables, the type of statistical analysis (Regr. = Regression; Corr. = Correlation), and the observed coefficients. Significance levels: * *p* < 0.05, ** *p* < 0.01, *** *p* < 0.001.

#### 3.2.7. Mindfulness

Mindfulness is the ability to be receptively open to sensory-motor, cognitive, and emotional stimuli and to be actively conscious of present events and experiences [105,106]. This definition of mindfulness implies the ability to accept events as they are, without judging them as either negative or positive [107]. Table 8 reports seven associations from five studies. The pattern is fully consistent in direction: all five associations with adverse mental-health outcomes are negative and significant, and both associations with quality of life and well-being are positive and significant. The regression coefficient for [82] (β = −2.48 ***, depression) is an unstandardized regression coefficient from a multiple-mediator model. All the studies that examined mindfulness assessed it via inferential analyses and considered it to be a dispositional resource in the transactional process of coping with cancer [82,83,108,109]. The evidence for mindfulness is consistent evidence as a dispositional resource.

**Table 8:** Associations of mindfulness and health outcomes in cancer survivors.

Role	Associated Variable	Type of Analysis	Coefficient	Study ID
	*Mental Health*			
-	Anxiety	Corr.	ρ_s_ = −0.50 ***	[110]
-	Depression	Corr.	ρ_s_ = −0.51	[110]
Dispo.	Depression	Regr.	β = −2.48 *** (unstandardized)	[82]
Dispo.	Psychological distress	Regr.	β = −0.36 **	[108]
-	Psychological symptoms	Corr.	r = −0.26 ***	[111]
	*Well-being and perceived health*			
Dispo.	Quality of Life	Regr.	β = 0.17 *	[109]
Dispo.	Well-Being	Regr.	β = 0.20 *	[83]

Legend. This table summarizes the findings from the systematic review regarding mindfulness. It details the theoretical role attributed to the resource (Dispo. = Dispositional), the associated mental health or quality of life variables, the type of statistical analysis (Regr. = Regression; Corr. = Correlation), and the observed coefficients. Coefficient marked “unstandardized” is raw regression coefficients (B) rather than standardized β, per the source study; its sign and significance remain interpretable, but its magnitude should not be compared directly to standardized estimates elsewhere in this table. Significance levels: * *p* < 0.05, ** *p* < 0.01, *** *p* < 0.001.

#### 3.2.8. Self-Compassion

According to Neff and Germer [112], self-compassion describes the ability to be touched by and open to one’s own suffering and to respond to it with kindness, understanding, and the desire to heal oneself. It involves looking after oneself in difficult situations, rather than falling into self-criticism, guilt, and self-pity. Table 9 reports ten associations from six studies (four using regression and two using correlation). The pattern is largely consistent: nine of ten associations point in the protective direction and are statistically significant. Self-compassion has a positive impact on cancer patients’ mental health by reducing stress [113] and symptoms of anxiety and depression [114,115,116], and by improving quality of life [109,114]. The one exception is [117], where self-compassion was significantly associated with lower anxiety (r = −0.19 **) but not with depression (r = −0.03, *ns*) in the same sample. Three of the four studies (75%) that examined self-compassion via inferential analyses considered it to be a dispositional resource in the transactional process of coping with cancer [109,113,115]. The remaining study (25%) considered it to be an evaluative resource [114]. The role attributed did not affect the direction or significance of the associations found. Self-compassion shows a consistent documented evidence base.

**Table 9:** Associations of Self-compassion and health outcomes in cancer survivors.

Role	Associated Variable	Type of Analysis	Coefficient	Study ID
	*Mental Health*			
Dispo.	Anxiety	Regr.	β = −0.41 ***	[115]
-	Anxiety	Corr.	r = −0.53 **	[116]
-	Anxiety	Corr.	r = −0.19 **	[117]
Eval.	Depression	Regr.	β = −0.54 **	[114]
Dispo.	Depression	Regr.	β = −0.34 ***	[115]
-	Depression	Corr.	r = −0.60 **	[116]
-	Depression	Corr.	r = −0.03	[117]
Dispo.	Stress	Regr.	β = −0.36 ***	[113]
	*Well-being and perceived health*			
Eval.	Quality of Life	Regr.	β = 0.40 ***	[114]
Dispo.	Quality of Life	Regr.	β = 0.43 ***	[109]

Legend. This table summarizes the findings from the systematic review regarding self-compassion. It details the theoretical role attributed to the resource (Dispo. = Dispositional; Eval. = Evaluative), the associated mental health or quality of life variables, the type of statistical analysis (Regr. = Regression; Corr. = Correlation), and the observed coefficients. Significance levels: ** *p* < 0.01, *** *p* < 0.001.

#### 3.2.9. Positive Affect

Positive affect is the propensity to experience positive emotional states [118]. Table 10 reports two associations, both from a single study [119]: significant negative associations with anxiety (β = −0.19 **) and depression (β = −0.16 **), consistent in direction with the pattern observed for other dispositional resources in this review. However, with only one study available, this evidence is preliminary; the resource’s protective association has not yet been independently replicated.

**Table 10:** Associations of positive affect and health outcomes in cancer survivors.

Role	Associated Variable	Type of Analysis	Coefficient	Study ID
	*Mental Health*			
Dispo.	Anxiety	MANCOVA	β = −0.19 **	[119]
Dispo.	Depression	MANCOVA	β = −0.16 **	[119]

Legend. This table summarizes the findings from the systematic review regarding positive affect. It details the theoretical role attributed to the resource (Dispo. = Dispositional), the associated mental health variables, the type of statistical analysis (MANCOVA = Multivariate Analysis of Covariance), and the observed coefficients. Significance levels: ** *p* < 0.01.

#### 3.2.10. Self-Efficacy

Self-efficacy describes one’s beliefs in one’s ability to carry out an action successfully, even in unpredictable and stressful situations [120,121,122]. Table 11 reports eight associations from five studies. The evidence here is markedly weaker than for the resources reviewed so far: only one of the four anxiety estimates reaches statistical significance, and one of three depression estimates does (β = −0.22 *** [73]). The one unambiguous finding is a significant positive association with quality of life (β = 0.37 *** [123]). Four of the five studies (80%) that evaluated self-efficacy considered it to be a dispositional resource in the transactional process of coping with cancer [33,66,73,124]. The remaining study (20%) considered it to be a coping resource [123]. Given the largely non-significant findings for anxiety and depression, self-efficacy’s evidence base rests almost entirely on its association with quality of life and requires further research before any claim of clinical utility.

**Table 11:** Associations of self-efficacy and health outcomes in cancer survivors.

Role	Associated Variable	Type of Analysis	Coefficient	Study ID
	*Mental Health*			
Dispo.	Anxiety	Regr.	β = −0.06	[73]
Dispo.	Anxiety	Regr.	β = 0.06	[66]
Dispo.	Anxiety	Corr.	r = −0.47 ***	[124]
Dispo.	Anxiety	Regr.	β = −0.06	[33]
Dispo.	Depression	Regr.	β = −0.22 ***	[73]
Dispo.	Depression	Regr.	β = −0.10	[66]
Dispo.	Depression	Regr.	β = −0.03	[33]
	*Well-being and perceived health*			
Cop.	Quality of life	Regr.	β = 0.37 ***	[123]

Legend. This table summarizes the findings from the systematic review regarding self-efficacy. It details the theoretical role attributed to the resource (Dispo. = Dispositional; Cop. = Coping), the associated mental health or quality of life variables, the type of statistical analysis (Regr. = Regression), and the observed coefficients. Significance levels: *** *p* < 0.001.

#### 3.2.11. Self-Esteem

Self-esteem describes how individuals perceive, judge, and value themselves [125]. Self-esteem has a positive impact on cancer patients’ mental health by reducing psychological distress [126]. Table 12 reports three associations, each from a different study. Only the association with psychological distress reaches significance (β = −0.27 ** [126], SEM coefficient). The depression association is non-significant (β = −0.17, *ns* [82]). The well-being association ([127], r = 0.54, *ns*) is reported without a significance level in the source. Two studies examined self-esteem via inferential analyses, with one (50%) considering it to be a dispositional resource in the transactional process of coping with cancer [82] and the other (50%) considering it an evaluative dimension [126]. With only one significant finding, self-esteem needs replication before any claim of clinical utility.

**Table 12:** Associations of self-esteem and health outcomes in cancer survivors.

Role	Associated Variable	Type of Analysis	Coefficient	Study ID
	*Mental Health*			
Dispo.	Depression	Regr.	β = −0.17	[82]
Eval.	Psychological distress	SEM	β = −0.27 **	[126]
	*Well-being and perceived health*			
-	Well-being	Corr.	r = 0.54	[127]

Legend. This table summarizes the findings from the systematic review regarding self-esteem. It details the theoretical role attributed to the resource (Dispo. = Dispositional; Eval. = Evaluative), the associated mental health or quality of life variables, the type of statistical analysis (Regr. = Regression; SEM = Structural Equation Modeling; Corr. = Correlation), and the observed coefficients. Significance levels: ** *p* < 0.01.

#### 3.2.12. Benefit-Finding

Benefit-finding is a common response to myriad threatening events. It predicts emotional and physical adaptation months, even years later [128]. Perceiving benefit in a bad situation gives meaning to suffering, which is why benefit-finding is considered a cognitive pathway toward post-traumatic growth [129]. Benefit-finding has a positive impact on cancer patients’ mental health in that it reduces symptoms of anxiety, depression, and psychological distress [81,130,131]. Table 13 reports six associations from five studies (four using regression and one using correlation). The pattern is consistent: four of six associations point in the protective direction and are statistically significant. 80% of the studies conceived benefit-finding as an evaluative resource in the transactional process of coping with cancer [81,130,131,132]. The remaining study (20%) did not define a specific role [133]. Benefit-finding shows a consistent direction but not enough documented evidence base, placing it among the resources warranting further research before systematic clinical integration.

**Table 13:** Associations of benefit-finding and health outcomes in cancer survivors.

Role	Associated Variable	Type of Analysis	Coefficient	Study ID
	*Mental Health*			
Eval.	Anxiety	Regr.	β = −0.51 *	[81]
Eval.	Depression	Regr.	β = −0.17 ***	[131]
Eval.	Depression	Regr.	β = −0.51 *	[81]
Eval.	Psychological distress	Regr.	β = −0.16 ***	[130]
Eval.	Psychological distress	Regr.	β = −0.15	[132]
	*Well-being and perceived health*			
-	Quality of Life	Corr.	r = −0.10	[133]

Legend. This table summarizes the findings from the systematic review regarding benefit-finding. It details the theoretical role attributed to the resource (Eval. = Evaluative), the associated mental health or quality of life variables, the type of statistical analysis (Regr. = Regression; Corr. = Correlation), and the observed coefficients. Significance levels: * *p* < 0.05, *** *p* < 0.001.

#### 3.2.13. Creativity

Creativity is the ability to produce work that is both novel and appropriate to its context [134]. Table 14 reports two associations, both from a single study [135]. Significant differences are observed for depression (z = 0.03 *), as well as for quality of life (F = 12.32 **). Potentially creative individuals display lower depression scores and higher quality of life compared to individuals with “blocked” creativity. The only study included in our review to examine creativity considered it to be a coping resource in the transactional process in cancer. However, in the absence of direct correlation or regression coefficients and given the small sample size (*n* = 35), this evidence remains preliminary; a causal or explanatory relationship has not yet been formally established. Creativity represents a promising resource whose value currently rests on theoretical and psycho-oncological relevance rather than robust empirical support, and its underlying mechanisms warrant further investigation through larger-scale replication studies.

**Table 14:** Associations of creativity and health outcomes in cancer survivors.

Role	Associated Variable	Type of Analysis	Coefficient	Study ID
	*Mental Health*			
Cop.	Depression	ANOVA	z = 0.03 *	[135]
	*Well-being and perceived health*			
Cop.	Quality of Life	ANOVA	F = 12.32 **	[135]

Legend. This table summarizes the findings from the systematic review regarding creativity. It details the theoretical role attributed to the resource (Cop. = Coping), the associated mental health or quality of life variables, the type of statistical analysis (ANOVA = Analysis of Variance), and the observed coefficients. Significance levels: * *p* < 0.05, ** *p* < 0.01.

#### 3.2.14. Self-Acceptance

Self-acceptance involves having a positive attitude toward the self, which includes good and bad qualities, and having positive feelings about the past [136]. Table 15 reports only one association from a single study [137], which found that self-acceptance had a positive impact on psychological symptoms and considered it to be a dispositional resource in the transactional process of coping with cancer. While self-acceptance shows potential as a protective factor, its role in psycho-oncology remains largely conceptual. Expanding this evidence base through larger cohorts will be essential to clarify its actual impact and clinical utility.

**Table 15:** Associations of self-acceptance and health outcomes in cancer survivors.

Role	Associated Variable	Type of Analysis	Coefficient	Study ID
	*Mental Health*			
Dispo.	Psychological symptoms	Regr.	β = −0.26 **	[137]

Legend. This table summarizes the findings from the systematic review regarding self-acceptance. It details the theoretical role attributed to the resource (Dispo. = Dispositional), the associated mental health variables, the type of statistical analysis (Regr. = Regression), and the observed coefficients. Significance levels: ** *p* < 0.01.

#### 3.2.15. Gratitude

Gratitude is a positive emotion aroused by receiving an intentional and selfless gift or help. It is linked to a feeling of connection with others, amplified perception of a situation’s positive aspects, and an increase in altruistic behaviors [138]. Table 16 reports two associations, both from a single study [139]. Gratitude has a positive effect on cancer patients’ mental health in that patients with higher levels of gratitude have lower levels of depression and anxiety [139]. Ruini and Vescovelli [139] considered gratitude to be a coping resource in the transactional process in cancer. The protective role of gratitude in cancer care remains largely theoretical, requiring larger replication studies to establish its true clinical impact.

**Table 16:** Associations of gratitude and health outcomes in cancer survivors.

Role	Associated Variable	Type of Analysis	Coefficient	Study ID
	*Mental Health*			
Cop.	Anxiety	ANOVA	F = 12.78 ***	[139]
Cop.	Depression	ANOVA	F = 4.16 ***	[139]

Legend. This table summarizes the findings from the systematic review regarding gratitude. It details the theoretical role attributed to the resource (Cop. = Coping), the associated mental health variables, the type of statistical analysis (ANOVA = Analysis of Variance), and the observed coefficients. Significance levels: *** *p* < 0.001.

#### 3.2.16. Meaning in Life

Wong [140] defined meaning in life in terms of four components—purpose, understanding, responsible action, and enjoyment/evaluation (PURE)—all of which are needed for life to have meaning. “Functionally, these components entail the four main types of psychological processes involved in having the good life: motivational (purpose, life goals, needs), cognitive (understanding, making sense of life), social/moral (responsibility, accountability, commitment), and affective (enjoyment/evaluation, positive emotions)” [140]. Table 17 reports only one association of one single study [137] which found that meaning in life reduced the psychological distress cancer patients feel. This study considered meaning in life to be an evaluative resource in the transactional process of coping with cancer. While meaning in life shows conceptual promise in psycho-oncology, empirical evidence from larger cohorts is still needed to validate its clinical utility.

**Table 17:** Associations of Meaning in life and health outcomes in cancer survivors.

Role	Associated Variable	Type of Analysis	Coefficient	Study ID
	*Mental Health*			
Eval.	Psychological symptoms	Regr.	β = −0.26 **	[137]

Legend. This table summarizes the findings from the systematic review regarding meaning in life. It details the theoretical role attributed to the resource (Eval. = Evaluative), the associated mental health variables, the type of statistical analysis (Regr. = Regression), and the observed coefficients. Significance levels: ** *p* < 0.01.

### 3.3. Quality of the Studies

The methodological quality and risk of bias of the 95 included studies were systematically appraised using the ROBINS-E tool. Overall, the quality of the studies was satisfactory (see Supplementary Material Table S6), with one study (1%) in the “low” risk of bias category, 88 studies (93%) in the “some concerns” category, and only six studies (6%) in the “high” risk of bias category.

Across the evaluated domains, the most prevalent methodological vulnerability was related to “confounding” domain. This was primarily driven by the predominantly cross-sectional nature of the included literature, where many studies lacked rigorous statistical adjustments for key clinical covariates such as cancer types, stages, or active treatments. Conversely, the structured data collection protocols within these cohorts minimized risks in the “post-exposure interventions” and “missing outcome data” domains, which were identified as the least common sources of bias.

### 3.4. Classification and Synthesis of Salutogenic Evidence

Reviewing these articles enabled us to identify each psychological resource’s salutogenic impacts on cancer patients’ mental health outcomes and to categorize them using a quantitative composite score mapping the robustness of the evidence. To ensure an objective and rigorous classification, the evidence level was determined by systematically combining three core criteria: (1) the nature of the statistical analysis conducted (ranging from a weight of 1 for unadjusted bivariate correlations to 3 for structural models, with standard inferential tests weighted at 2), (2) the overall risk of bias appraised via the ROBINS-E tool (weighted from 4 for low risk to 1 for critical risk), and (3) the study volume (ranging from a multiplier of 1.0 for *n* = 1 to 4 for *n* = 7), rewarding consistency and replication across the literature. The resulting empirical scores, which ranged from 2.00 to 20.50, were segmented into specific thresholds to define the shading intensity of our heatmap and determine the final evidence categories (see Table 18):

- Convergent evidence (score ≥ 13.00): this category reflects evidence supported by superior methodological quality (low-to-moderate risk of bias), advanced statistical analyses, or high study replication. At this level, strong specific associations are observed for pairings such as optimism and depression (20.50), hope and anxiety (18.00), or resilience and quality of life (18.00).
- Supported and indicative evidence (13.00 > Score ≥ 6.00): this threshold highlights established trends characterized by acceptable methodological quality or standard inferential statistics, though sometimes limited by lower study replication or smaller sample sizes. Examples include self-compassion and depression (12.00), gratitude and anxiety (6.00) or spirituality and quality of life (10.00).
- Emergent evidence (score < 6.00): this category denotes preliminary findings typically relying on unadjusted correlations or higher risks of bias, requiring further replication to establish a definitive role. This trend is confined to specific intersections, such as positive affect and depression (4.00), mindfulness and anxiety (2.00) or self-esteem and well-being (3.00).
- Lack of evidence: applied when primary data were entirely absent or insufficient to calculate a composite score, signaling unexamined associations in the current psycho-oncology literature (e.g., creativity and anxiety).

**Table 18:** Heatmap of Psychological Resources’ Positive Impacts on Health Outcomes.

Psychological Resource	Health Outcomes				
	Anxiety	Depression	Psychological Distress	Quality of Life	Well-Being
Resilience	16.00	15.27	12.00	18.00	4.00
Post-Traumatic Growth	12.00	13.50	6.00	8.00	
Hope	18.00	18.18	4.00	8.00	
Optimism	18.29	20.50		12.00	4.00
Sense of coherence	10.00	15.00		18.50	
Spirituality	2.00	6.67		10.00	
Mindfulness	2.00	8.00	3.00	4.00	4.00
Self-Compassion	6.67	12.00	4.00	10.00	
Positive affect	4.00	4.00			
Self-Efficacy	15.00	9.33		4.00	
Self-Esteem		6.00	9.00		3.00
Benefit finding	4.00	8.00	12.00	2.00	
Creativity		4.00		4.00	
Self-Acceptance			4.00		
Gratitude	6.00	6.00			
Meaning in Life			4.00		

Legend. Shading intensity represents the robustness of evidence supporting the positive impact of each resource on mental health outcomes based on a composite score: Green (Score ≥ 13.00): Strong and convergent evidence. Yellow (13.00 > Score ≥ 6.00): Moderate/indicative evidence. Red (6.00 > Score): Emergent evidence. White: Lack of evidence or insufficient data.

Second, an overarching resource-level synthesis was performed to map the general trends of the literature, moving beyond individual resource-to-outcome pairings to evaluate each psychological resource’s overall impact for cancer survivors. Resources were categorized into profiles based on the overall distribution and consistency of their specific scores:

- Consolidated Impact Profile: This profile highlights resources demonstrating a consistent and well-supported positive trend across multiple dimensions of mental health. These resources are characterized by a favorable balance between superior study quality (low-to-moderate risk of bias), advanced statistical testing, and a substantial volume of independent research replications. The psychological resources exhibiting this empirical convergence across the literature are optimism, hope, resilience, and sense of coherence.
- Emerging Impact Profile: This profile captures resources displaying informative trends or localized efficacy, but whose cumulative evidence requires further methodological consolidation. The current literature for these resources is characterized by more heterogeneous study quality, smaller clusters, or a predominance of simpler, unadjusted statistical models. The resources into this category are post-traumatic growth, spirituality, self-compassion, gratitude and self-efficacy.
- Frontier Impact Profile: This profile encompasses emerging psychological resources that show conceptual promise and encouraging preliminary findings, yet currently represent a smaller footprint in the empirical literature. The existing body of work for these resources is characterized by a limited number of dedicated studies or initial exploratory frameworks. The resources into this category are mindfulness, benefit-finding, positive affect, self-esteem, self-acceptance, meaning in life, and creativity.

## 4. Discussion

The current systematic literature review aimed to shed light on the roles psychological resources play in helping adult patients cope with cancer. By analyzing recent research on this issue, we were able to determine which resources have been studied most frequently and the impact of these resources on adult cancer patients’ mental health. In addition, we used the multifactorial and transactional model of health to ascertain the type of role that each psychological resource plays. Our findings provide a better understanding of these key issues and the potential for mobilizing them to provide support to patients.

The studies included in the current review examined just 16 of the 72 psychological resources described in the literature. A first observation warranting discussion is the sheer number of protective factors in the context of cancer. This abundance highlights both the true conceptual diversity of psychological resources and the ongoing need for clearer theoretical boundaries, as several concepts still tend to overlap. This profusion also carries a practical implication: when so many resources are potentially relevant, clinicians and intervention designers are left with limited guidance on which to prioritize. In other words, research relating to cancer has been limited to a small number of psychological resources, notably the best-defined and most-clearly conceptualized resources for which validated evaluation tools are available in several languages. These resources include resilience, optimism, hope, and post-traumatic growth. In fact, the definitions of some psychological resources are vague, multiple, non-consensual, and difficult to operationalize [141]. The last 15 years have seen growing interest in resources such as self-compassion, spirituality, and mindfulness, which correspond to the concept of transcendence, that is the bringing together of the “strengths that forge connections to the larger universe and provide meaning” [142]. Peterson and Seligman’s [142] systematic review showed that most research up to this time had focused on the links between psychological resources and unfavorable health outcomes such as anxiety, depression, and psychological distress. Conversely, as noted by Schmidt et al. [143], a lack of research made it difficult to ascertain the limits of psychological resources’ positive outcomes on quality of life and well-being and the conditions required to obtain these positive outcomes.

Our review also revealed large differences in the amount of empirical proof regarding the links between psychological resources and adult cancer patients’ mental health outcomes. The vulnerability and uncertainty associated with cancer have a profound impact on cancer patients, changing their self-image and body satisfaction, and leading them to question their identity and sense of continuity [144]. Self-esteem, meaning in life, and self-acceptance could reduce this negative impact by enabling cancer patients to reinterpret the adverse situation [145,146,147], but a lack of research on these resources’ salutogenic potential means that more evidence of their positive effects is needed before integrating them into clinical interventions. In addition, health psychologists long studied personality traits (e.g., type C) as pathogenic factors, but this perspective recently fell out of favor and has been replaced by studies of coping factors. The rise of positive psychology and improvements in life expectancy with cancer has led to renewed interest in dispositional factors, especially salutogenic resources.

More research is needed to better determine the contributions of resources situated within the “Frontier Impact Profile”. This profile includes mindfulness, benefit-finding, positive affect, self-esteem, self-acceptance, meaning in life, and creativity, for which the current volume and consistency of evidence remain insufficient to draw conclusions. Although there is some evidence for the salutogenic function of post-traumatic growth, spirituality, self-compassion, gratitude and self-efficacy, the level of certainty attached to these associations remains limited, corresponding to the “Emerging Impact Profile” identified in our risk-of-bias-weighted synthesis.

In contrast, the positive impacts of optimism, resilience, hope, and sense of coherence are much more established, corresponding to the “Consolidated Impact Profile” derived from the synthesis, which combines a higher volume of studies, greater consistency of statistically significant findings, and a more favorable risk-of-bias appraisal. By translating this extensive inventory into a hierarchy of evidentiary strength, this classification offers a practical framework for prioritization—guiding clinical attention toward resources with the most robust support (Consolidated) as promising targets for interventions and care pathways, while identifying those requiring further investigation before clinical application (Emerging and Frontier). Finally, classifying psychological resources in this manner points toward a possible need for clearer operational definitions.

Analysis of the studies included in the current review showed substantial differences in how researchers conceptualize some psychological resources’ roles in coping with cancer. Nevertheless, a majority of studies converged on the role played by certain resources, with most studies that examined resilience, post-traumatic growth, hope, optimism, sense of coherence, mindfulness, self-compassion, positive affect, self-efficacy, and self-acceptance considering them to be dispositional resources that directly impact mental health outcomes. Hence, these studies assessed these resources’ trait dimensions without considering their state dimensions, even though their state dimensions are described in the literature. Similarly, majority of studies that examined benefit-finding, meaning in life, and self-esteem considered them to be evaluative resources that impact the way patients assess both their situation and themselves, and majority of studies that examined creativity, gratitude, and spirituality considered them to be coping resources, mobilized within emotion-centered coping strategies.

Despite the generally satisfactory quality of the studies, it is important to consider potential biases when interpreting their results. As confounding was the most prevalent risk identified, particular care must be taken when evaluating how the results were selected and reported. Conversely, the least frequent risk was “bias due to missing outcome data,” which shows that the studies managed missing data well. This spread of risks of bias highlights the need to consider results critically, especially when the presentation of results might have been influenced by publication bias or selective reporting. Indeed, psychology research as a whole has faced a crisis of confidence in recent years, with numerous questions being asked about its credibility [148], mostly due to poor reproducibility of results, serious methodological biases, and misuse of statistical practices in studies published in peer-reviewed journals, including those with high impact factors. Fortunately, proven cases of scientific fraud, such as fabricating or falsifying data, are rare [149]. This crisis results from the convergence of several systemic problems, notably: (1) research protocols based on samples that are too small to give satisfactory statistical power; (2) a reluctance to submit articles for publication when the study did not produce statistically significant results for fear of the article being rejected by editorial boards; (3) non-meticulous research practices due to the pressure to publish, which prioritizes quantity over quality; (4) the trend towards publishing ever-more concise accounts that do not provide enough details to satisfactorily evaluate a study; and (5) a lack of transparency due to the failure to report all data and full details of the analyses methods used.

**Figure 2:**
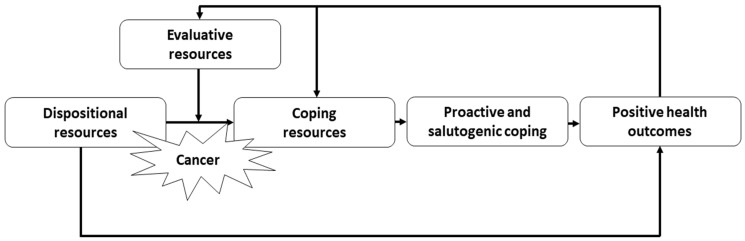
Proposed conceptual framework for Psychological Resources for Coping with Cancer (PRCC). Legend. This model illustrates how dispositional resources influence the appraisal of cancer as a stressor. The experience of cancer triggers the mobilization of evaluative and coping resources. While evaluative resources determine how the cancer experience is perceived, coping resources facilitate the transition toward proactive and salutogenic strategies. Feedback loops indicate that positive health outcomes reinforce both evaluative and coping resources, creating a dynamic process of adaptation.

Notwithstanding these methodological limitations inherent to the field, synthesizing the available empirical evidence remains essential to advance conceptual clarity. To this end, we drew on the results of our systematic review to inductively define a theoretical model of how adult cancer patients mobilize the three different types of psychological resources. Rather than a definitive causal map, the Psychological Resources for Coping with Cancer (PRCC) model (see Figure 2) is presented as a hypothesis-generating framework grounded in the empirical relationships identified across the included studies. This model is a conceptual proposition derived from the integration and discussion of our findings, rather than a direct empirical result. The architecture of the PRCC model was derived directly from the role classification established in the Section 3 (Table 2, Table 3, Table 4, Table 5, Table 6, Table 7, Table 8, Table 9, Table 10, Table 11, Table 12, Table 13, Table 14, Table 15, Table 16 and Table 17), rather than from the strength-of-evidence heatmap. In doing so, the model addresses the conceptual fragmentation noted above by organizing the initially large and heterogeneous set of resources into a small number of functionally distinct pathways, rather than treating them as a flat list of independent factors. Resources for which most included studies converged on a conceptual role—dispositional (resilience, post-traumatic growth, hope, optimism, sense of coherence, mindfulness, self-compassion, positive affect, self-efficacy, self-acceptance), evaluative (self-esteem, benefit-finding, meaning in life,), or coping (spirituality, creativity, gratitude)—were used to define the model’s three structural pathways. The model suggests that dispositional resources may directly reduce psychological symptoms and help patients develop or maintain favorable health outcomes such as well-being and quality of life. But dispositional resources also act indirectly by activating coping resources during cancer diagnosis and treatment. Evaluative resources help patients to consider the disease objectively and logically, which leads them to mobilize coping resources that favor favorable health outcomes by activating one or more independent pathways [150,151]: the physiological stress-response mechanism, proactive and salutogenic coping strategies, and social resources (family, friends, caregivers, etc.). These assets favor positive health outcomes by activating proactive and salutogenic strategies, consistent with a dynamic adaptation process.

### 4.1. Limitations

Our review’s first limitation arises from the choice of terms for the literature search, as the versatility of the terms chosen inevitably resulted in the identification of a very large number of cancer-related studies. Some of these terms are used in contexts outside the field of psychological resources, sometimes simply to highlight the positive nature of results (e.g., hope for a new drug-based treatment). This polysemy undoubtedly extended the screening process by initially including studies that had no direct relevance to the evaluation of psychological resources.

Including all sites, types, and stages of cancer is another potential limitation, as these factors greatly impact patients’ subjective experiences of cancer. For example, a patient being diagnosed with stage 1 cancer has a very different experience and mobilizes different resources to a patient whose cancer has metastasized. This inherent heterogeneity may limit the external validity of our findings when applied to specific oncological sub-populations, as certain resources might be pivotal depending on the prognosis or treatment toxicity. However, the criterion that participants had to be undergoing treatment for cancer attenuates this potential limitation by not including studies involving cancer patients in remission or candidates for screening.

A narrative synthesis was conducted rather than a quantitative meta-analysis due to the substantial methodological and clinical heterogeneity observed across the included studies. Specifically, the diversity of psychometric instruments used to assess psychological resources, the wide range of cancer types and stages, and the inconsistent reporting of statistical formats (e.g., varying use of correlation coefficients, regression weights, and ANOVA values) precluded a robust and reliable pooling of effect sizes.

Furthermore, potential publication bias and search bias must be considered. In the field of psycho-oncology, studies demonstrating significant salutogenic effects are more likely to be published than those showing no impact, which may lead to an overestimation of some resources’ benefits. Nevertheless, the diversity of outcomes observed in our 95 included studies suggests a broad representation of the current literature. Additionally, our search was restricted to English- and French- language publications, which may have excluded relevant studies published in other languages. This limitation is partially attenuated by the near-equal representation of Western and Asian populations among the included studies, suggesting that our findings are not confined to a single cultural or regional context. Nevertheless, this linguistic restriction may still have introduced a degree of regional or cultural selection bias that cannot be fully ruled out.

Finally, our review was strictly restricted to observational quantitative designs, deliberately excluding qualitative and interventional studies. While this choice was methodologically necessary to allow for standardized comparisons of statistical relationships and to observe ‘spontaneous’ resource dynamics, it inherently limits the depth of our findings. Qualitative research offers invaluable, idiographic insights into the subjective, lived experience of mobilizing psychological resources that quantitative metrics cannot fully capture. Consequently, our findings map statistical patterns across populations, highlighting the need for future qualitative syntheses to complement this quantitative blueprint.

### 4.2. Perspectives

Future research modeling the ways in which patients cope with cancer should adopt a positive psychology perspective and fully integrate the role of psychological resources. It is essential to develop and validate specific tools for evaluating these resources in health contexts. To avoid, for example, unrealistic positive expectations in the face of poor prognoses, particular care must be paid to incorporate somatic aspects when considering a prognosis, the debilitating effects of cancer, and the disease’s social consequences. Detailed analysis of action mechanisms is also needed. Although the current review identified the resources associated with the best outcomes, it is essential to study in greater detail how these resources impact the progression of the disease and patients’ responses to treatment, quality of life and psychological well-being. This will require longitudinal studies, interventions to strengthen patients’ psychological resources, and research on the underlying biological or behavioral pathways. It will also be necessary to identify specific populations (types of cancer, stages of the disease, age, gender, etc.) for whom certain resources are more important, so interventions can be tailored to each patient’s needs. Finally, study of interactions between psychological resources is fundamental, as these resources contribute jointly to coping with cancer. Detailed statistical analyses could shed light on these dynamics.

From an applied perspective, the objective is to complement the currently dominant deficit model of health by integrating psychological resources into health interventions. However, the inherent heterogeneity of cancer populations—encompassing diverse malignancies and stages—dictates that such integration must not be a ‘one-size-fits-all’ approach. This will require developing and evaluating therapeutic programs to help cancer patients build self-compassion and resilience, resources whose salutogenic function is well established. The aim would also be to promote the emergence of positive effects and realistic levels of hope, optimism, gratitude, and self-compassion. Developing mindfulness is another promising avenue. Integrating these approaches, arising from positive health psychology, will fill a gap not only in the field of cancer prevention, but also by promoting optimum health states for cancer patients.

## 5. Conclusions

In addition to highlighting the heterogeneity of current research and the need for a more integrative approach to studying psychological resources, the current systematic review enabled us to inductively draw up a hypothesis-generating model (Psychological Resources in Coping with Cancer—PRCC) of how psychological resources act and interact to help adult patients cope with cancer. Beyond providing initial data supporting the theoretical foundations of the PRCC model, the review’s findings lay the groundwork for research programs designed to empirically test its different stages. Ultimately, this initiative aims to offer a renewed perspective in positive health psychology—one that places equal emphasis on psychological resources and vulnerabilities to help adult cancer patients and individuals at high risk of cancer develop favorable health outcomes.

## 6. Patents

**Protocol registration**: The protocol for this systematic review has been registered in the PROSPERO registry under No.: CRD420251021051.

## Figures and Tables

**Table 1:** Number of studies and participants for each cancer site at diagnosis.

Site at Diagnosis	Studies		Participants	
	*N*	%	*N*	%
Breast	26	27%	6468	30%
Digestive	14	15%	4389	20%
Gynecological	7	7%	1336	6%
Prostate	4	4%	1660	8%
Brain	3	3%	538	2%
Blood	2	2%	447	2%
Head and neck	2	2%	240	1%
Lung	2	2%	492	2%
Rare	2	2%	406	2%
Urological	1	1%	284	1%
All sites/Unspecified	32	34%	5613	26%

## Data Availability

Data sharing not applicable to this article as no datasets were generated or analyzed during the current study.

## Supplementary Materials

The following supporting information can be downloaded at: https://www.mdpi.com/article/10.3390/cancers18152525/s1, Table S1: Full list of search terms; Table S2: Draft search strategies for all databases; Table S3: Instruments Used to Assess the Health Outcomes Studied; Table S4: Instruments Used to Assess the Psychological Resources Studied; Table S5: Main characteristics of the included studies (*N* = 95); Table S6: ROBINS-E Risk of Bias Assessments for the Included Studies; PRISMA 2020 checklist [152].
