# Supplementary material for: Psychological Resources in Adults with Cancer: A Systematic Review of Quantitative Studies
Source: Cancers (Basel). 2026 Aug 6;18(15):2525. doi: 10.3390/cancers18152525 (PMC13465765; doi:10.3390/cancers18152525)
Supplement: Supplementary file 1 [file cancers-18-02525-s001.zip › cancers-4382083-supplementary - after proof - email - reset refs.pdf]

SUPPLEMENTARY TABLE S1

Full list of search terms

Cancer / Oncological Disease :

- Cancer*
- Tumour*
- Oncolog*
- Carcinoma
- Malignan*
- Neoplasm*
- Metasta*
- Sarcoma*
- Leukaemia
- Lymphoma
- Mesothelioma
- Myeloma
- Myelodysplastic

Altruism And Interpersonal Skills

- Altruis*
- Compassion
- Empathy
- Forgiveness
- Generosity
- Gratitude
- Helpfulness
- Interpersonal Ability*
- Interpersonal Skill*
- Kindness
- Niceness
- Social Abilit*
- Social Competence*

Authenticity And Self-Esteem

- Authenticity
- Honest*
- Integrity
- Loyalty
- Modesty
- Self-Acceptance
- Self-Esteem
- Self-Worth

Cognitive Resources

- Cognitive Resource*
- Sense Of Coherence
- Learning Ability*
- Learning Skill*
- Positive Reinterpretation

Bravery And Perseverance

- Bravery
- Confidence
- Courag*
- Persistence
- Self-Confidence
- Toughness
- Trust
- Vigor
- Vitality
- Character Strength*
- Fortitude
- Hardiness

Creativity, Flourishing, And Positive Emotions

- Creativ*
- Curiosity
- Flourishing
- Flow
- Ingenuity
- Originality
- Playfulness
- Enthusiasm
- Humo*R
- Self-Realization
- Thriving
- Zest
- Happiness

Positive Affect And General Resources

- Positive Affect*
- Resourceful*
- Valor
- Adaptive Resource*
- Individual Resource*
- Internal Resource*
- Personal Resource*
- Psychological Resource*
- Psychosocial Resource*

Meaning, Mindfulness, And OpenMindedness

- Sense Of Meaning
- Meaning Making
- Meaning-Making
- Mindfulness
- Open-Mindedness
- Conscientiousness
- Openness

Spirituality And Beliefs

- Faith
- Religio*
- Spiritual*
- Transcenden*

Hope, Post-Traumatic Growth, And Resilience

- Future Orientation
- Future-Mindedness
- Hope*
- Optimism
- Purpose In Life
- Purposiveness
- Sense Of Purpose
- Willpower
- Adversarial Growth
- Benefit Finding
- Benefit-Finding
- Finding Benefits
- Personal Benefits
- Personal Growth
- Positive Growth
- Posttraumatic Growth
- Post-Traumatic Growth
- Resilien*
- Stress-Related Growth

SUPPLEMENTARY TABLE S2

Draft search strategies for all databases

MEDLINE

1. (cancer* OR tumour* OR oncolog* OR carcinoma OR malignan* OR neoplasm* OR metasta* OR sarcoma* OR leukaemia OR lymphoma OR mesothelioma OR myeloma OR myelodysplastic).ti,ab. AND (Altruis* OR compassion OR Empathy OR forgiveness OR generosity OR Gratitude OR Helpfulness OR "Interpersonal ability*" OR "Interpersonal skill*" OR Kindness OR Niceness OR "Social abilit*" OR "social competence*").ti,ab.
2. (cancer* OR tumour* OR oncolog* OR carcinoma OR malignan* OR neoplasm* OR metasta* OR sarcoma* OR leukaemia OR lymphoma OR mesothelioma OR myeloma OR myelodysplastic).ti,ab. AND (Authenticity OR honest* OR Integrity OR Loyalty OR Modesty OR "Self-acceptance" OR "self-esteem" OR "self-worth").ti,ab.
3. (cancer* OR tumour* OR oncolog* OR carcinoma OR malignan* OR neoplasm* OR metasta* OR sarcoma* OR leukaemia OR lymphoma OR mesothelioma OR myeloma OR myelodysplastic).ti,ab. AND (Bravery OR confidence OR Courag* OR Persistence OR "self-confidence" OR Toughness OR Trust OR Vigor OR Vitality OR "character strength*" OR fortitude OR hardiness).ti,ab.
4. (cancer* OR tumour* OR oncolog* OR carcinoma OR malignan* OR neoplasm* OR metasta* OR sarcoma* OR leukaemia OR lymphoma OR mesothelioma OR myeloma OR myelodysplastic).ti,ab. AND ("Cognitive resource*" OR "sense of coherence" OR "Learning ability*" OR "Learning skill*" OR "positive reinterpretation").ti,ab.
5. (cancer* OR tumour* OR oncolog* OR carcinoma OR malignan* OR neoplasm* OR metasta* OR sarcoma* OR leukaemia OR lymphoma OR mesothelioma OR myeloma OR myelodysplastic).ti,ab. AND (Creativ* OR Curiosity OR flourishing OR flow OR Ingenuity OR Originality OR Playfulness OR enthusiasm OR Humo*r OR "self-realization" OR thriving OR Zest OR happiness).ti,ab.
6. (cancer* OR tumour* OR oncolog* OR carcinoma OR malignan* OR neoplasm* OR metasta* OR sarcoma* OR leukaemia OR lymphoma OR mesothelioma OR myeloma OR myelodysplastic).ti,ab. AND ("positive affect*" OR Resourceful* OR Valor OR "Adaptive resource*" OR "Individual resource*" OR "Internal resource*" OR "Personal resource*" OR "Psychological resource*" OR "Psychosocial resource*").ti,ab.
7. (cancer* OR tumour* OR oncolog* OR carcinoma OR malignan* OR neoplasm* OR metasta* OR sarcoma* OR leukaemia OR lymphoma OR mesothelioma OR myeloma OR myelodysplastic).ti,ab. AND ("sense of meaning" OR "Meaning making" OR "meaning-making" OR Mindfulness OR "openmindedness" OR conscientiousness OR Openness).ti,ab.
8. (cancer* OR tumour* OR oncolog* OR carcinoma OR malignan* OR neoplasm* OR metasta* OR sarcoma* OR leukaemia OR lymphoma OR mesothelioma OR myeloma OR myelodysplastic).ti,ab. AND ("future orientation" OR "future-mindedness" OR Hope* OR optimism OR "purpose in life" OR purposiveness OR "sense of purpose" OR Willpower OR "adversarial growth" OR "benefit finding" OR "benefit-finding" OR "finding benefits" OR "personal benefits" OR "personal growth" OR "positive growth" OR "posttraumatic growth" OR "post-traumatic growth" OR Resilien* OR "stress-related growth").ti,ab.
9. (cancer* OR tumour* OR oncolog* OR carcinoma OR malignan* OR neoplasm* OR metasta* OR sarcoma* OR leukaemia OR lymphoma OR mesothelioma OR myeloma OR myelodysplastic).ti,ab. AND (Faith OR Religio* OR Spiritual* OR Transcenden*).ti,ab.

PSYCINFO

1. AB,TI(cancer* OR tumour* OR oncolog* OR carcinoma OR malignan* OR neoplasm* OR metasta* OR sarcoma* OR leukaemia OR lymphoma OR mesothelioma OR myeloma OR myelodysplastic) AND AB,TI(Altruis* OR compassion OR Empathy OR forgiveness OR generosity OR Gratitude OR Helpfulness
OR "Interpersonal ability*" OR "Interpersonal skill*" OR Kindness OR Niceness OR "Social abilit*" OR "social competence*")
2. AB,TI(cancer* OR tumour* OR oncolog* OR carcinoma OR malignan* OR neoplasm* OR metasta* OR sarcoma* OR leukaemia OR lymphoma OR mesothelioma OR myeloma OR myelodysplastic) AND
AB,TI(Authenticity OR honest* OR Integrity OR Loyalty OR Modesty OR Self-acceptance OR self-esteem OR self-worth)
3. AB,TI(cancer* OR tumour* OR oncolog* OR carcinoma OR malignan* OR neoplasm* OR metasta* OR sarcoma* OR leukaemia OR lymphoma OR mesothelioma OR myeloma OR myelodysplastic) AND AB,TI(Bravery OR confidence OR Courag* OR Persistence OR self-confidence OR Toughness OR Trust OR Vigor OR Vitality OR "character strength*" OR fortitude OR hardiness)
4. AB,TI(cancer* OR tumour* OR oncolog* OR carcinoma OR malignan* OR neoplasm* OR metasta* OR sarcoma* OR leukaemia OR lymphoma OR mesothelioma OR myeloma OR myelodysplastic) AND
AB,TI("Cognitive resource*" OR "sense of coherence" OR "Learning ability*" OR "Learning skill*" OR "positive reinterpretation")
5. AB,TI(cancer* OR tumour* OR oncolog* OR carcinoma OR malignan* OR neoplasm* OR metasta* OR sarcoma* OR leukaemia OR lymphoma OR mesothelioma OR myeloma OR myelodysplastic) AND AB,TI(Creativ* OR Curiosity OR flourishing OR flow OR Ingenuity OR Originality OR Playfulness OR enthusiasm OR Humo*r OR self-realization OR thriving OR Zest OR happiness)
6. AB,TI(cancer* OR tumour* OR oncolog* OR carcinoma OR malignan* OR neoplasm* OR metasta* OR sarcoma* OR leukaemia OR lymphoma OR mesothelioma OR myeloma OR myelodysplastic) AND AB,TI("positive affect*" OR Resourceful* OR Valor OR "Adaptive resource*" OR "Individual resource*" OR "Internal resource*" OR "Personal resource*" OR "Psychological resource*" OR "Psychosocial resource*")
7. AB,TI(cancer* OR tumour* OR oncolog* OR carcinoma OR malignan* OR neoplasm* OR metasta* OR sarcoma* OR leukaemia OR lymphoma OR mesothelioma OR myeloma OR myelodysplastic) AND AB,TI("sense of meaning" OR "Meaning making" OR meaning-making OR Mindfulness OR openmindedness OR conscientiousness OR Openness)
8. AB,TI(cancer* OR tumour* OR oncolog* OR carcinoma OR malignan* OR neoplasm* OR metasta* OR sarcoma* OR leukaemia OR lymphoma OR mesothelioma OR myeloma OR myelodysplastic) AND AB,TI("future orientation OR future-mindedness OR Hope* OR optimism OR "purpose in life" OR purposiveness OR "sense of purpose" OR Willpower OR "adversarial growth" OR "benefit finding" OR benefit-finding OR "finding benefits" OR "personal benefits" OR "personal growth" OR "positive growth" OR "posttraumatic growth" OR "post-traumatic growth" OR Resilien* OR "stress-related growth")
9. AB,TI(cancer* OR tumour* OR oncolog* OR carcinoma OR malignan* OR neoplasm* OR metasta* OR sarcoma* OR leukaemia OR lymphoma OR mesothelioma OR myeloma OR myelodysplastic) AND AB,TI(Faith OR Religio* OR Spiritual* OR Transcenden*)

PUBMED

1. (cancer* [tiab] OR tumour* [tiab] OR oncolog* [tiab] OR carcinoma [tiab] OR malignan* [tiab] OR neoplasm* [tiab] OR metasta* [tiab] OR sarcoma* [tiab] OR leukaemia [tiab] OR lymphoma [tiab] OR mesothelioma [tiab] OR myeloma [tiab] OR myelodysplastic [tiab]) AND (Altruis* [tiab] OR compassion [tiab] OR Empathy [tiab] OR forgiveness [tiab] OR generosity [tiab] OR Gratitude [tiab] OR Helpfulness [tiab] OR "Interpersonal ability*" [tiab] OR "Interpersonal skill*" [tiab] OR Kindness [tiab] OR Niceness [tiab] OR "Social abilit*" [tiab] OR "social competence*" [tiab])
2. (cancer* [tiab] OR tumour* [tiab] OR oncolog* [tiab] OR carcinoma [tiab] OR malignan* [tiab] OR neoplasm* [tiab] OR metasta* [tiab] OR sarcoma* [tiab] OR leukaemia [tiab] OR lymphoma [tiab] OR mesothelioma [tiab] OR myeloma [tiab] OR myelodysplastic [tiab]) AND (Authenticity [tiab] OR honest* [tiab] OR Integrity [tiab] OR Loyalty [tiab] OR Modesty [tiab] OR "Self-acceptance" [tiab] OR "selfesteem" [tiab] OR "self-worth" [tiab])
3. (cancer* [tiab] OR tumour* [tiab] OR oncolog* [tiab] OR carcinoma [tiab] OR malignan* [tiab] OR neoplasm* [tiab] OR metasta* [tiab] OR sarcoma* [tiab] OR leukaemia [tiab] OR lymphoma [tiab] OR mesothelioma [tiab] OR myeloma [tiab] OR myelodysplastic [tiab]) AND (Bravery [tiab] OR confidence [tiab] OR Courag* [tiab] OR Persistence [tiab] OR "self-confidence" [tiab] OR Toughness [tiab] OR Trust [tiab] OR Vigor [tiab] OR Vitality [tiab] OR "character strength*" [tiab] OR fortitude [tiab] OR hardiness [tiab])
4. (cancer* [tiab] OR tumour* [tiab] OR oncolog* [tiab] OR carcinoma [tiab] OR malignan* [tiab] OR neoplasm* [tiab] OR metasta* [tiab] OR sarcoma* [tiab] OR leukaemia [tiab] OR lymphoma [tiab] OR mesothelioma [tiab] OR myeloma [tiab] OR myelodysplastic [tiab]) AND ("Cognitive resource*" [tiab] OR "sense of coherence" [tiab] OR "Learning ability*" [tiab] OR "Learning skill*" [tiab] OR "positive reinterpretation" [tiab])
5. (cancer* [tiab] OR tumour* [tiab] OR oncolog* [tiab] OR carcinoma [tiab] OR malignan* [tiab] OR neoplasm* [tiab] OR metasta* [tiab] OR sarcoma* [tiab] OR leukaemia [tiab] OR lymphoma [tiab] OR mesothelioma [tiab] OR myeloma [tiab] OR myelodysplastic [tiab]) AND (Creativ* [tiab] OR Curiosity [tiab] OR flourishing [tiab] OR flow [tiab] OR Ingenuity [tiab] OR Originality [tiab] OR Playfulness [tiab] OR enthusiasm [tiab] OR Humo*r [tiab] OR "self-realization" [tiab] OR thriving [tiab] OR Zest [tiab] OR happiness [tiab])
6. (cancer* [tiab] OR tumour* [tiab] OR oncolog* [tiab] OR carcinoma [tiab] OR malignan* [tiab] OR neoplasm* [tiab] OR metasta* [tiab] OR sarcoma* [tiab] OR leukaemia [tiab] OR lymphoma [tiab] OR mesothelioma [tiab] OR myeloma [tiab] OR myelodysplastic [tiab]) AND ("positive affect*" [tiab] OR Resourceful* [tiab] OR Valor [tiab] OR "Adaptive resource*" [tiab] OR "Individual resource*" [tiab] OR "Internal resource*" [tiab] OR "Personal resource*" [tiab] OR "Psychological resource*" [tiab] OR "Psychosocial resource*" [tiab])
7. (cancer* [tiab] OR tumour* [tiab] OR oncolog* [tiab] OR carcinoma [tiab] OR malignan* [tiab] OR neoplasm* [tiab] OR metasta* [tiab] OR sarcoma* [tiab] OR leukaemia [tiab] OR lymphoma [tiab] OR mesothelioma [tiab] OR myeloma [tiab] OR myelodysplastic [tiab]) AND ("sense of meaning" [tiab] OR "Meaning making" [tiab] OR "meaning-making" [tiab] OR Mindfulness [tiab] OR "open-mindedness" [tiab] OR conscientiousness [tiab] OR Openness [tiab])
8. (cancer* [tiab] OR tumour* [tiab] OR oncolog* [tiab] OR carcinoma [tiab] OR malignan* [tiab] OR neoplasm* [tiab] OR metasta* [tiab] OR sarcoma* [tiab] OR leukaemia [tiab] OR lymphoma [tiab] OR mesothelioma [tiab] OR myeloma [tiab] OR myelodysplastic [tiab]) AND ("future orientation" [tiab] OR "future-mindedness" [tiab] OR Hope* [tiab] OR optimism [tiab] OR "purpose in life" [tiab] OR purposiveness [tiab] OR "sense of purpose" [tiab] OR Willpower [tiab] OR "adversarial growth" [tiab] OR "benefit finding" [tiab] OR "benefit-finding" [tiab] OR "finding benefits" [tiab] OR "personal benefits" [tiab] OR "personal growth" [tiab] OR "positive growth" [tiab] OR "posttraumatic growth" [tiab] OR "post-traumatic growth" [tiab] OR Resilien* [tiab] OR "stress-related growth" [tiab])
9. (cancer* [tiab] OR tumour* [tiab] OR oncolog* [tiab] OR carcinoma [tiab] OR malignan* [tiab] OR neoplasm* [tiab] OR metasta* [tiab] OR sarcoma* [tiab] OR leukaemia [tiab] OR lymphoma [tiab] OR

mesothelioma [tiab] OR myeloma [tiab] OR myelodysplastic [tiab]) AND (Faith [tiab] OR Religio* [tiab] OR Spiritual* [tiab] OR Transcenden* [tiab])

ScienceDirect and SpringerLink

1. TITLE-ABS-KEY(cancer* OR tumour* OR oncolog* OR carcinoma OR malignan* OR neoplasm* OR metasta* OR sarcoma* OR leukaemia OR lymphoma OR mesothelioma OR myeloma OR myelodysplastic) AND TITLE-ABS-KEY(Altruis* OR compassion OR Empathy OR forgiveness OR generosity
   OR Gratitude OR Helpfulness OR "Interpersonal ability*" OR "Interpersonal skill*" OR Kindness OR Niceness OR "Social abilit*" OR "social competence*")
2. TITLE-ABS-KEY(cancer* OR tumour* OR oncolog* OR carcinoma OR malignan* OR neoplasm* OR metasta* OR sarcoma* OR leukaemia OR lymphoma OR mesothelioma OR myeloma OR myelodysplastic) AND TITLE-ABS-KEY(Authenticity OR honest* OR Integrity OR Loyalty OR Modesty OR "Self-acceptance" OR "self-esteem" OR "self-worth")
3. TITLE-ABS-KEY(cancer* OR tumour* OR oncolog* OR carcinoma OR malignan* OR neoplasm* OR metasta* OR sarcoma* OR leukaemia OR lymphoma OR mesothelioma OR myeloma OR myelodysplastic) AND TITLE-ABS-KEY(Bravery OR confidence OR Courag* OR Persistence OR "selfconfidence" OR Toughness OR Trust OR Vigor OR Vitality OR "character strength*" OR fortitude OR hardiness)
4. TITLE-ABS-KEY(cancer* OR tumour* OR oncolog* OR carcinoma OR malignan* OR neoplasm* OR metasta* OR sarcoma* OR leukaemia OR lymphoma OR mesothelioma OR myeloma OR myelodysplastic) AND TITLE-ABS-KEY("Cognitive resource*" OR "sense of coherence" OR "Learning ability*" OR "Learning skill*" OR "positive reinterpretation")
5. TITLE-ABS-KEY(cancer* OR tumour* OR oncolog* OR carcinoma OR malignan* OR neoplasm* OR metasta* OR sarcoma* OR leukaemia OR lymphoma OR mesothelioma OR myeloma OR myelodysplastic) AND TITLE-ABS-KEY(Creativ* OR Curiosity OR flourishing OR flow OR Ingenuity OR Originality OR Playfulness OR enthusiasm OR Humo*r OR "self-realization" OR thriving OR Zest OR happiness)
6. TITLE-ABS-KEY(cancer* OR tumour* OR oncolog* OR carcinoma OR malignan* OR neoplasm* OR metasta* OR sarcoma* OR leukaemia OR lymphoma OR mesothelioma OR myeloma OR myelodysplastic) AND TITLE-ABS-KEY("positive affect*" OR Resourceful* OR Valor OR "Adaptive resource*" OR "Individual resource*" OR "Internal resource*" OR "Personal resource*" OR "Psychological resource*" OR "Psychosocial resource*")
7. TITLE-ABS-KEY(cancer* OR tumour* OR oncolog* OR carcinoma OR malignan* OR neoplasm* OR metasta* OR sarcoma* OR leukaemia OR lymphoma OR mesothelioma OR myeloma OR myelodysplastic) AND TITLE-ABS-KEY("sense of meaning" OR "Meaning making" OR "meaning-making" OR Mindfulness OR "open-mindedness" OR conscientiousness OR Openness)
8. TITLE-ABS-KEY(cancer* OR tumour* OR oncolog* OR carcinoma OR malignan* OR neoplasm* OR metasta* OR sarcoma* OR leukaemia OR lymphoma OR mesothelioma OR myeloma OR myelodysplastic) AND TITLE-ABS-KEY("future orientation" OR "future-mindedness" OR Hope* OR optimism OR "purpose in life" OR purposiveness OR "sense of purpose" OR Willpower OR "adversarial growth" OR "benefit finding" OR "benefit-finding" OR "finding benefits" OR "personal benefits" OR "personal growth" OR "positive growth" OR "posttraumatic growth" OR "post-traumatic growth" OR Resilien* OR "stress-related growth")
9. TITLE-ABS-KEY(cancer* OR tumour* OR oncolog* OR carcinoma OR malignan* OR neoplasm* OR metasta* OR sarcoma* OR leukaemia OR lymphoma OR mesothelioma OR myeloma OR myelodysplastic) AND TITLE-ABS-KEY(Faith OR Religio* OR Spiritual* OR Transcenden*)

Wiley Online Library

1. ALL(cancer* OR tumour* OR oncolog* OR carcinoma OR malignan* OR neoplasm* OR metasta* OR sarcoma* OR leukaemia OR lymphoma OR mesothelioma OR myeloma OR myelodysplastic) AND ALL(Altruis* OR compassion OR Empathy OR forgiveness OR generosity OR Gratitude OR Helpfulness OR "Interpersonal ability*" OR "Interpersonal skill*" OR Kindness OR Niceness OR "Social abilit*" OR "social competence*")
2. ALL(cancer* OR tumour* OR oncolog* OR carcinoma OR malignan* OR neoplasm* OR metasta* OR sarcoma* OR leukaemia OR lymphoma OR mesothelioma OR myeloma OR myelodysplastic) AND ALL(Authenticity OR honest* OR Integrity OR Loyalty OR Modesty OR "Self-acceptance" OR "selfesteem" OR "self-worth")
3. ALL(cancer* OR tumour* OR oncolog* OR carcinoma OR malignan* OR neoplasm* OR metasta* OR sarcoma* OR leukaemia OR lymphoma OR mesothelioma OR myeloma OR myelodysplastic) AND ALL(Bravery OR confidence OR Courag* OR Persistence OR "self-confidence" OR Toughness OR Trust OR Vigor OR Vitality OR "character strength*" OR fortitude OR hardiness)
4. ALL(cancer* OR tumour* OR oncolog* OR carcinoma OR malignan* OR neoplasm* OR metasta* OR sarcoma* OR leukaemia OR lymphoma OR mesothelioma OR myeloma OR myelodysplastic) AND ALL("Cognitive resource*" OR "sense of coherence" OR "Learning ability*" OR "Learning skill*" OR "positive reinterpretation")
5. ALL(cancer* OR tumour* OR oncolog* OR carcinoma OR malignan* OR neoplasm* OR metasta* OR sarcoma* OR leukaemia OR lymphoma OR mesothelioma OR myeloma OR myelodysplastic) AND ALL(Creativ* OR Curiosity OR flourishing OR flow OR Ingenuity OR Originality OR Playfulness OR enthusiasm OR Humo*r OR "self-realization" OR thriving OR Zest OR happiness)
6. ALL(cancer* OR tumour* OR oncolog* OR carcinoma OR malignan* OR neoplasm* OR metasta* OR sarcoma* OR leukaemia OR lymphoma OR mesothelioma OR myeloma OR myelodysplastic) AND ALL("positive affect*" OR Resourceful* OR Valor OR "Adaptive resource*" OR "Individual resource*" OR "Internal resource*" OR "Personal resource*" OR "Psychological resource*" OR "Psychosocial resource*")
7. ALL(cancer* OR tumour* OR oncolog* OR carcinoma OR malignan* OR neoplasm* OR metasta* OR sarcoma* OR leukaemia OR lymphoma OR mesothelioma OR myeloma OR myelodysplastic) AND ALL("sense of meaning" OR "Meaning making" OR "meaning-making" OR Mindfulness OR "openmindedness" OR conscientiousness OR Openness)
8. ALL(cancer* OR tumour* OR oncolog* OR carcinoma OR malignan* OR neoplasm* OR metasta* OR sarcoma* OR leukaemia OR lymphoma OR mesothelioma OR myeloma OR myelodysplastic) AND ALL("future orientation" OR "future-mindedness" OR Hope* OR optimism OR "purpose in life" OR purposiveness OR "sense of purpose" OR Willpower OR "adversarial growth" OR "benefit finding" OR "benefit-finding" OR "finding benefits" OR "personal benefits" OR "personal growth" OR "positive growth" OR "posttraumatic growth" OR "post-traumatic growth" OR Resilien* OR "stress-related growth")
9. ALL(cancer* OR tumour* OR oncolog* OR carcinoma OR malignan* OR neoplasm* OR metasta* OR sarcoma* OR leukaemia OR lymphoma OR mesothelioma OR myeloma OR myelodysplastic) AND ALL(Faith OR Religio* OR Spiritual* OR Transcenden*)

SUPPLEMENTARY TABLE S3

Instruments Used to Assess *the Health Outcomes Studied*

**Health outcome**	**Instruments**	**N**
Anxiety	Hospital Anxiety and Depression Scale	22
	Zung Self-Rating Anxiety Scale	5
	State and Trait Anxiety Inventory	4
	Death Anxiety Scale	1
	Endler Multidimensional Anxiety Scales	1
	Fear of Progression Questionnaire-Short Form	1
	General Anxiety Disorder Scale	1
Basic trust	Basic Trust Inventory	1
Depression	Hospital Anxiety and Depression Scale	24
	Center for Epidemiologic Studies Depression Scale	9
	Patient Health Questionnaire	6
	Beck Depression Inventory	3
	Hamilton Depression Scale	1
	Siddiqui–Shah Depression Scale	1
Insomnia	Insomnia Severity index	1
Psychological distress	Depression, Anxiety and Stress Scale	4
	Distress Thermometer	3
	Perceived Stress Scale	2
	M.D. Anderson Symptom Inventory	1
	Profile of Mood States Short Form	1
	Symptom Distress Checklist	1
Post-Traumatic Stress Disorder	Impact of Event Scale	1
	PTSD checklist for DSM-5	1
Quality of life	European Organization for Research and Treatment of Cancer Quality of Life Questionnaire Core 30	12
	Functional Assessment of Cancer Therapy-General Scale	7
	World Health Organization Quality of Life	5
	SF-36	2
	EQ-5D-3L visual analogue scale	1
	Numeric Scale	1
Well-being	General Health Questionnaire	4
	Satisfaction with Life Questionnaire	1
	Items: "Considering your life in general, how happy would you describe yourself?"; "How happy would you describe yourself at this moment"	1

SUPPLEMENTARY TABLE S4

Instruments Used to Assess the Psychological Resources Studied

Psychological resource	Instruments	N
Benefit-finding	Benefit Finding Scale	4
Creativity	Big Five Inventory Corsi	1<br>1
Gratitude	Gratitude Questionnaire	1
Hope	Adult Hope Scale<br>Herth Hope Index<br>Herth Hope Scale<br>Adult Dispositional Hope Scale	5<br>5<br>2<br>1
Meaning in life	Meaning in life Questionnaire	1
Mindfulness	Five Facet Mindfulness Questionnaire-Short Form Mindfulness<br>Attention Awareness Scale	3<br>3
Optimism	Life Orientation Test<br>Life Orientation Scale-Revised<br>Optimism–Pessimism Scale<br>Orientation to Life Questionnaire	13<br>4<br>1<br>1
Positive affect	Positive and Negative Affective Schedule	4
Post-traumatic growth	Post-traumatic Growth Inventory<br>Life Changing Events Scale	16<br>1
Resilience	Connor-Davidson Psychological Resilience Scale<br>Resilience Scale<br>Brief Resilience Scale<br>Kremen and Block's Ego Resiliency Scale<br>Wagnild and Young's Resilience Scale	14<br>8<br>1<br>1<br>1
Self-acceptance	Self-Acceptance Questionnaire	1
Self-compassion	Self-Compassion Scale	6
Self-efficacy	General Self-Efficacy Scale<br>General Perceived Self-Efficacy Scale<br>Self-Efficacy for Advanced Cancer	2<br>1<br>1
Self-esteem	Rosenberg Self-esteem Scale Positivity<br>Scale	3<br>1
Sense of coherence	Sense Of Coherence Scale	12
Spirituality	Functional Assessment of Chronic Illness Therapy –Spiritual Well-Being Scale<br>Spiritual Coping Questionnaire<br>Spiritual Perspective Scale<br>Spiritual and Religious Attitudes in Dealing with Illness	4<br>1<br>1<br>1

Note. NR: Not Reported. Study Design: comp.: comparative. Metastatic Status: Mixed : stages I–IV; Metastatic : stage IV.

SUPPLEMENTARY TABLE S5

Main characteristics of the included studies ($N = 95$).

Study id.	Author	Date	Country	Study design	Setting	Sample size	Age mean (SD)	N female (%)	Localisation	Metastatic	Resources investigated	Health issues
[130]	Liu	2018	China	Cross-sectional	Mixed	351	57.34 (9.05)	167 (48%)	All Localisations	Non-metastatic	Benefit finding	Distress
[131]	Manne	2018	USA	Cross-sectional	Outpatients	174	55.32 (10.28)	174 (100%)	Gynecological	Mixed	Benefit finding	Depression
[81]	Mo	2022	China	Cross-sectional	Outpatients	512	47.46 (8.51)	512 (100%)	Breast	Non-metastatic	Optimism; Benefit finding	Anxiety; Depression
[132]	Zimmaro	2021	USA	Longitudinal	Outpatients	133	57.1 (13.3)	40 (31%)	Colorectal	Mixed	Benefit finding	Psychological distress
[133]	Herts	2021	USA; Australia	Longitudinal	Outpatients	107	58.96 (12.84)	49 (46%)	Uveal Melanoma	NR	Posttraumatic growth	Depression
[135]	Delpech	2021	France	Cross sectional (comp.)	Inpatients	35	61 (11)	19 (54%)	NR	NR	Creativity	Depression; Quality of life
[139]	Ruini	2013	Italy	Cross-sectional	Outpatients	67	56.6 (11.7)	67 (100%)	Breast	Non-metastatic	Posttraumatic growth; Gratitude	Anxiety; Depression
[65]	Abdollahi	2019	Iran	Cross-sectional	Outpatients	220	46.23 (8.8)	220 (100%)	Breast	Mixed	Hope	Stress
[66]	Bao	2017	China	Cross-sectional	NR	222	50.16 (13.57)	129 (58%)	NR	NR	Hope; Self-efficacy; Optimism	Anxiety; Depression
[67]	Berendes	2010	USA	Cross-sectional	Mixed	51	65 (8.56)	43 (84%)	Lung	NR	Hope	Depression
[68]	Chang	2014	Taïwan	Longitudinal (comp.)	Outpatients	67	NR	30 (45%)	Breast	NR	Hope	Anxiety; Depression; Quuality of life
[69]	Hou	2010	China	Longitudinal	Inpatients	234	64.44 (10.55)	89 (38%)	Colorectal	Metastatic	Hope; Optimism	Anxiety; Depression
[32]	Liu	2017	China	Cross-sectional	Inpatients	198	55.71 (9.03)	198 (100%)	Ovarian	Mixed	Hope; Resilience	Anxiety; Depression
[70]	Mayer	2021	Germany	Cross sectional (comp.)	Inpatients	66	53.7 (11.1)	30 (45%)	Brain	Mixed	Hope	Depression
[71]	Peh	2017	Singapore	Cross-sectional	Outpatients	144	48.96 (9.23)	94 (65%)	All Localisations	NR	Hope	Anxiety; Depression

Study id.	Author	Date	Country	Study design	Setting	Sample size	Age mean (SD)	N female (%)	Localisation	Metastatic	Resources investigated	Health issues
[72]	Tao	2022	China	Cross-sectional	Mixed	442	52.03 (12.14)	161 (36%)	All Localisations	NR	Hope; Spirituality	Anxiety; Depression
[73]	Yang	2014	China	Cross-sectional	Inpatients	224	49.16 (10.11)	224 (100%)	Cervical	Mixed	Hope; Self-efficacy; Optimism	Anxiety; Depression
[74]	Zarzycka	2019	Poland	Cross-sectional	Inpatients	77	52 (10.97)	77 (100%)	All Localisations	NR	Hope	Anxiety
[28]	Zhao	2021	China	Cross-sectional	Inpatients	564	59.66 (11.21)	0 (0%)	Prostate	Non-metastatic	Hope; Resilience	Depression
[47]	Liu	2018	China	Cross-sectional	NR	284	56.86 (11.38)	106 (37%)	Renal	Non-metastatic	Hope; Optimism; Resilience	Quality of life
[75]	van der Biessen	2017	Netherlands	Cross-sectional	Outpatients	135	61.8 (10.3)	70 (52%)	All Localisations	Metastatic	Hope	Quality of life
[76]	Yang	2016	China	Cross-sectional	Outpatients	489	59.1 (11.28)	144 (29%)	Bladder ; Kidney	Mixed	Hope; Optimism	Anxiety; Depression
[137]	Zhou	2019	China	Cross-sectional	Outpatients	292	52.2 (11.8)	NR	Gastrointestinal	Mixed	Self-Acceptance	Anxiety; Depression
[82]	Kuhlman	2017	USA	Cross-sectional	Outpatients	271	53.23 (11.49)	271 (100%)	Breast	Non-metastatic	Optimism; Self-esteem; Mindfulness	Depression
[108]	Omid	2017	Iran	Cross-sectional	Outpatients	109	NR	77 (71%)	All Localisations	NR	Posttraumatic growth; Mindfulness	Anxiety; Depression
[109]	Garcia	2021	Brazil	Cross-sectional	Outpatients	183	62.8 (12.7)	98 (54%)	All Localisations	NR	Self-compassion; Mindfulness	Quality of life
[83]	Pama	2018	Netherlands	Longitudinal	Outpatients	120	63.5 (10.5)	47 (39%)	Colorectal	Mixed	Optimism; Mindfulness	Well-being
[110]	Zimmaro	2020	USA	Cross-sectional	Outpatients	64	57.3 (11.4)	100 (156%)	Breast	Metastatic	Mindfulness	Anxiety; Depression
[111]	Xu	2017	USA; China; Netherlands	Cross-sectional	Outpatients	176	55.7 (11.22)	81 (46%)	Gastrointestinal	Mixed	Mindfulness	Anxiety; Depression
[79]	Sumpio	2017	USA	Cross-sectional	Outpatients	121	NR	61 (50%)	All Localisations	Mixed	Optimism	Psychological distress
[84]	Abbas	2022	Pakistan	Cross-sectional	Outpatients	200	26.6 (NR)	100 (50%)	NR	Mixed	Optimism	Anxiety; Depression

Study id.	Author	Date	Country	Study design	Setting	Sample size	Age mean (SD)	N female (%)	Localisation	Metastatic	Resources investigated	Health issues
[78]	Gustavsson-Lilius	2012	Finland	Longitudinal	Mixed	147	58 (8.7)	81 (55%)	All Localisations	Mixed	Sense of coherence; Optimism	Anxiety; Depression
[85]	MacDonald	2021	USA	Longitudinal (comp.)	Outpatients	299	NR	NR	Uveal Melanoma	NR	Optimism	Depression
[33]	Wang	2016	China	Cross-sectional	Inpatients	227	45.48 (16.05)	110 (48%)	Hematological	Mixed	Self-efficacy; Optimism; Resilience	Anxiety; Depression
[114]	Pinto-Gouveia	2014	Portugal	Cross-sectional	Outpatients	63	NR	52 (83%)	All Localisations	NR	Self-compassion	Depression; Quality of life
[80]	Caprara	2016	Italy	Longitudinal	Outpatients	100	59.62 (10.33)	58 (58%)	Colon; Lung ; Breast	Mixed	Optimism; Self-esteem	Quality of life
[86]	Yoon	2015	Korea	Cross-sectional	Outpatients	339	NR	122 (36%)	Colorectal ; Stomach ; Lung ; Liver ; Kidney	Mixed	Optimism	Quality of life
[87]	Finck	2018	Germany; Colombia	Cross-sectional	Mixed	462	55.7 (11.5)	95 (21%)	Breast	NR	Optimism	Quality of life
[119]	Hou	2010	China	Longitudinal (comp.)	Outpatients	215	64.28 (10.58)	82 (38%)	Colorectal	Mixed	Positive Affect	Anxiety; Depression
[59]	Tomita	2017	Japan	Cross-sectional	Outpatients	157	59.08 (10.06)	157 (100%)	Breast	Mixed	Posttraumatic growth	Depression
[62]	Chan	2011	China	Cross-sectional	Outpatients	170	48.36 (7)	170 (100%)	Breast	NR	Posttraumatic growth	PTSD
[52]	Gundogmus	2022	Turkey	Cross-sectional	Outpatients	71	50.52 (9.04)	71 (100%)	Breast	NR	Posttraumatic growth; Resilience	Anxiety; Depression; PTSD
[60]	Li	2016	China	Cross-sectional	Mixed	198	57.70 (12.11)	93 (47%)	All Localisations	Mixed	Posttraumatic growth	Anxiety; Depression
[51]	Liu	2020	China	Cross-sectional	Outpatients	612	46.8 (13.1)	612 (100%)	Breast	Mixed	Posttraumatic growth	Anxiety; Depression
[50]	Silva	2012	Portugal		Inpatients	50	52.1 (8.3)	50 (100%)	Breast	Non-metastatic	Posttraumatic growth	Anxiety; Depression; Quality of life
[58]	Tomich	2012	USA	Longitudinal	Outpatients	72	62.58 (10.32)	36 (50%)	Colorectal ; Lung	Metastatic	Posttraumatic growth	Quality of life
[53]	Abdullah	2015	Malaysia	Longitudinal	Outpatients	50	49.76 (11.56)	34 (68%)	Head And Neck	Mixed	Posttraumatic growth	Anxiety; Depression

Study id.	Author	Date	Country	Study design	Setting	Sample size	Age mean (SD)	N female (%)	Localisation	Metastatic	Resources investigated	Health issues
[54]	Bourdon	2019	France	Longitudinal	Mixed	293	52.5 (9.9)	246 (84%)	Breast ; Melanoma	NR	Posttraumatic growth	Anxiety; Depression; Quality of life
[55]	Danhauer	2013	USA	Longitudinal	Mixed	653	54 (NR)	653 (100%)	Breast	Non-metastatic	Spirituality; Optimism; Posttraumatic growth	Quality of life
[56]	Nik Jaafar	2022	Malaysia	Cross-sectional	Outpatients		NR	NR	Head And Neck	NR	Posttraumatic growth	Anxiety
[57]	Paredes	2018	Portugal	Cross-sectional	Outpatients	100	52.45 (11.93)	100 (100%)	Breast	NR	Spirituality; Posttraumatic growth	Psychological distress
[41]	Matzka	2016	Austria	Cross-sectional	Mixed	343	58 (14.4)	202 (59%)	All Localisations	Mixed	Resilience	Psychological distress
[26]	Gan	2018	China	Longitudinal	Inpatients	307	58.35 (11.32)	159 (52%)	All Localisations	Mixed	Posttraumatic growth; Resilience	Anxiety; Depression
[61]	Villanova Quiroga	2020	Brazil	Cross-sectional	Outpatients	84	55.3 (12.7)	84 (100%)	Breast	Mixed	Posttraumatic growth	Depression
[63]	Trzebiński	2013	Poland	Cross-sectional	Outpatients	61	NR	37 (61%)	All Localisations	NR	Posttraumatic growth	Basic trust
[40]	Chang	2020	Taiwan	Longitudinal	Mixed	42	54.5 (8.9)	42 (100%)	Endometrial	Mixed	Resilience	Psychological distress
[39]	Er	2022	Turquie	Cross-sectional	Outpatients	105	53.22 (13.46)	77 (73%)	All Localisations	Non-metastatic	Resilience	Distress
[38]	Vaughan	2018	Australia	Cross-sectional	Outpatients	227	64.57 (11.61)	115 (51%)	All Localisations	Mixed	Resilience	Psychological distress
[27]	Ye	2016	China	Cross-sectional	Outpatients	276	NR	NR	Lung; Gastrointestinal ; Colorectal	Mixed	Resilience	Anxiety; Depression; Quality of life
[29]	Zahid	2021	Pakistan	Cross-sectional	Outpatients	250	NR	81 (32%)	Brain	NR	Resilience	Anxiety; Depression
[31]	Markovitz	2015	Belgium ; Netherlands	Cross-sectional (comp.)	Inpatients	464	53.9 (10.5)	253 (55%)	Breast	NR	Resilience	Anxiety; Depression
[37]	Sharpley	2021	Australia	Cross-sectional	Outpatients	576	69.17 (6.91)	0 (0%)	Prostate	NR	Resilience	Depression
[34]	Sharpley	2014	Australia	Cross sectional (comp.)	Outpatients	425	70.193 (6.35)	0 (0%)	Prostate	NR	Resilience	Depression

Study id.	Author	Date	Country	Study design	Setting	Sample size	Age mean (SD)	N female (%)	Localisation	Metastatic	Resources investigated	Health issues
[35]	Lee	2021	Republic of Korea	Longitudinal	Outpatients	193	44.89 (7.913)	193 (100%)	Breast	Non-metastatic	Resilience	Anxiety; Depression; Quality of life
[30]	Li	2016	China	Cross-sectional	Outpatients	591	63.77 (11.75)	63 (11%)	Bladder ; Renal	Non-metastatic	Resilience	Anxiety; Depression
[36]	Martin	2021	USA	Cross-sectional	Mixed	95	67 (9)	0 (0%)	Prostate	Mixed	Resilience	Anxiety; Depression
[44]	Macía	2020	Spain	Cross-sectional	Community-based	74	50.9 (NR)	59 (80%)	All Localisations	NR	Resilience	Quality of life
[45]	Macía	2021	Spain	Cross-sectional	Outpatients	170	49 (NR)	134 (79%)	All Localisations	Mixed	Resilience	Quality of life
[42]	Manne	2015	USA; China	Cross-sectional	Outpatients	281	54.8 (10.5)	281 (100%)	Gynecological	Mixed	Resilience	Quality of life
[42]	Manne	2018	Germany; Austria	Cross-sectional	Outpatients	304	57.34 (14.5)	177 (58%)	All Localisations	Mixed	Resilience	Quality of life
[46]	Abdollahi	2022	Iran	Cross-sectional	Community-based	195	45.32 (8.2)	195 (100%)	Breast	Mixed	Resilience	Quality of life
[25]	Lv	2023	China	Cross-sectional	Inpatients	366	58.51 (11.98)	119 (33%)	Gastrointestinal	Non-metastatic	Resilience	Insomnia
[113]	Abdollahi	2020	Iran	Cross-sectional	Community-based	210	NR	210 (100%)	Breast	Metastatic	Self-compassion	Stress
[115]	Zhu	2019	China; Netherlands	Longitudinal	Outpatients	153	50.78 (11.61)	100 (65%)	All Localisations	Mixed	Self-compassion	Anxiety; Depression
[116]	Sherman	2017	Australia	Cross-sectional	Mixed	75	47.81 (8.86)	75 (100%)	Breast	NR	Self-compassion	Anxiety; Depression
[117]	Wei	2022	China	Cross-sectional	Mixed	289	50.11 (13.05)	180 (62%)	All Localisations	Mixed	Self-compassion	Anxiety; Depression
[123]	Sato	2015	Japan	Cross-sectional	Outpatients	156	NR	NR	All Localisations	NR	Self-efficacy	Quality of life
[124]	Mystakidou	2010	Greece	Cross-sectional	Mixed	99	63.5 (13.2)	59 (60%)	All Localisations	Mixed	Self-efficacy	Anxiety
[126]	Tian	2021	China	Cross-sectional	Outpatients	441	59.48 (9.93)	126 (29%)	Lung	Mixed	Self-esteem	Psychological distress
[127]	Cobo-Cuenca	2019	Spain	Cross-sectional	Community-based	514	46.34 (8.276)	514 (100%)	Breast	Non-metastatic	Self-esteem	Well-being
[92]	Kim	2021	Republic of Korea	Cross-sectional	Inpatients	148	52.17 (10.66)	148 (100%)	Gynecological	Mixed	Sense of coherence	Depression; Quality of life
[98]	Li	2023	China	Cross-sectional	Mixed	269	53.45 (9.35)	269 (100%)	Cervical	Mixed	Sense of coherence	Anxiety; Depression
[99]	Wei	2019	China	Cross-sectional	Mixed	575	42.72 (11.99)	566 (98%)	Breast	Mixed	Sense of coherence	Depression

Study id.	Author	Date	Country	Study design	Setting	Sample size	Age mean (SD)	N female (%)	Localisation	Metastatic	Resources investigated	Health issues
[97]	Hyphantis	2011	Greece	Longitudinal	Outpatients	144	65.1 (9.8)	47 (33%)	Colorectal	Non-metastatic	Sense of coherence	Quality of life; Psychological distress
[93]	Paika	2010	Greece	Cross-sectional	Outpatients	162	65 (9.5)	53 (33%)	Colorectal	Mixed	Sense of coherence	Quality of life
[94]	Rohani	2015	Iran	Longitudinal (comp.)	Inpatients	372	46.1 (9.8)	162 (44%)	Breast	Mixed	Spirituality; Sense of coherence	Quality of life
[95]	Rohani	2015	Iran	Longitudinal	Inpatients	162	46.1 (9.8)	162 (100%)	Breast	Non-metastatic	Sense of coherence	Quality of life
[96]	Sarenmalm	2013	Sweden	Longitudinal	Mixed	131	65 (NR)	131 (100%)	Breast	NR	Sense of coherence	Quality of life
[90]	Asplund	2017	Sweden ; Denmark	Cross-sectional	Outpatients	1085	NR	490 (45%)	Rectal	Mixed	Sense of coherence	Quality of life
[91]	Qiu	2020	China	Cross-sectional	Outpatients	162	59.7 (10.95)	73 (45%)	All Localisations	Metastatic	Sense of coherence	Quality of life
[89]	Drott	2020	Sweden	Cross-sectional	Outpatients	43	62 (8)	36 (84%)	Colorectal	NR	Sense of coherence	Quality of life
[101]	Leeson	2015	USA	Longitudinal	Outpatients	220	NR	84 (38%)	Hematological	NR	Spirituality	Well-being
[102]	Bai	2018	USA	Cross-sectional	Outpatients	102	55.2 (9.6)	63 (62%)	All Localisations	Mixed	Spirituality	Quality of life
[103]	Bovero	2016	Italy	Cross-sectional	Inpatients	115	67.06 (12.3)	37 (32%)	All Localisations	Mixed	Spirituality	Well-being
[104]	Gaston-Johansson	2013	USA	Cross-sectional	Outpatients	17	50.12 (10.73)	17 (100%)	Breast	Non-metastatic	Spirituality; Sense of coherence	Anxiety; Depression

Note. NR: Not Reported. Study Design: comp.: comparative. Metastatic Status: Mixed : stages I–IV; Metastatic : stage IV ; Metastatic : stages I-III.

SUPPLEMENTARY TABLE S6

ROBINS-E Risk of Bias Assessments for the Included Studies

**Study id.**	**Domain 1: Bias due to confounding**	**Domain 2: Bias arising from measurement of the exposure**	**Domain 3: Bias in selection of participants into the study (or analysis)**	**Domain 4: Bias due to post-exposure interventions**	**Domain 5: Bias due to missing data**	**Domain 6: Bias arising from measurement of the outcome**	**Domain 7: Bias in selection of the reported result**	**Global ROBINS-E**
[130]	High risk	Some concerns	Low risk	Low risk	Low risk	Some concerns	Some concerns	Some concerns
[131]	High risk	Some concerns	Some concerns	Low risk	Low risk	Some concerns	Low risk	Some concerns
[81]	High risk	Some concerns	Some concerns	Low risk	Low risk	Some concerns	Some concerns	Some concerns
[132]	Some concerns	Some concerns	Some concerns	Low risk	Some concerns	Some concerns	Some concerns	Some concerns
[133]	High risk	Some concerns	Some concerns	Low risk	Some concerns	Some concerns	Some concerns	Some concerns
[135]	High risk	High risk	Some concerns	Low risk	Low risk	Some concerns	Some concerns	High risk
[139]	High risk	Some concerns	Some concerns	Low risk	Low risk	Some concerns	Some concerns	Some concerns
[65]	High risk	Some concerns	Some concerns	Low risk	Some concerns	Some concerns	Some concerns	Some concerns
[66]	Some concerns	Some concerns	Some concerns	Low risk	Low risk	Some concerns	Some concerns	Some concerns
[67]	High risk	Some concerns	Some concerns	Low risk	Some concerns	Some concerns	Some concerns	Some concerns
[68]	High risk	Some concerns	Some concerns	Some concerns	Some concerns	Some concerns	Some concerns	Some concerns
[69]	Some concerns	Some concerns	Some concerns	Low risk	Some concerns	Some concerns	Low risk	Some concerns
[32]	High risk	Some concerns	Some concerns	Some concerns	Low risk	Some concerns	Some concerns	Some concerns
[70]	High risk	High risk	Some concerns	Low risk	Low risk	Some concerns	Some concerns	High risk
[71]	High risk	Some concerns	Some concerns	Low risk	Low risk	Some concerns	Some concerns	Some concerns
[72]	High risk	Some concerns	Some concerns	Low risk	Low risk	Some concerns	Some concerns	High risk
[73]	High risk	Some concerns	Some concerns	Some concerns	Some concerns	Some concerns	Some concerns	Some concerns
[74]	High risk	Some concerns	Some concerns	Low risk	Low risk	Some concerns	Some concerns	Some concerns
[28]	High risk	Some concerns	Some concerns	Low risk	Low risk	Some concerns	Some concerns	Some concerns
[47]	High risk	Some concerns	Some concerns	Some concerns	Low risk	Some concerns	Some concerns	Some concerns
[75]	Some concerns	Some concerns	Some concerns	Low risk	Low risk	Some concerns	Some concerns	Some concerns
[76]	High risk	Some concerns	Some concerns	Low risk	Low risk	Some concerns	Some concerns	Some concerns
[137]	High risk	Some concerns	Some concerns	Low risk	Low risk	Some concerns	Some concerns	Some concerns

Study id.	Domain 1: Bias due to confounding	Domain 2: Bias arising from measurement of the exposure	Domain 3: Bias in selection of participants into the study (or analysis)	Domain 4: Bias due to post-exposure interventions	Domain 5: Bias due to missing data	Domain 6: Bias arising from measurement of the outcome	Domain 7: Bias in selection of the reported result	Global ROBINS-E
[82]	Some concerns	Some concerns	Some concerns	Low risk	Low risk	Some concerns	Low risk	Some concerns
[108]	High risk	Some concerns	Some concerns	Low risk	Low risk	Some concerns	Some concerns	Some concerns
[109]	High risk	High risk	Some concerns	Some concerns	Low risk	Some concerns	Some concerns	High risk
[83]	High risk	Some concerns	Some concerns	Low risk	Some concerns	Some concerns	Some concerns	Some concerns
[110]	High risk	Some concerns	Some concerns	Low risk	Low risk	Some concerns	Some concerns	Some concerns
[111]	Some concerns	Some concerns	Some concerns	Low risk	Low risk	Some concerns	Some concerns	Some concerns
[79]	High risk	Some concerns	Some concerns	Low risk	Low risk	Some concerns	Some concerns	Some concerns
[84]	High risk	Some concerns	Some concerns	Low risk	Some concerns	Some concerns	Some concerns	Some concerns
[78]	Some concerns	Some concerns	Low risk	Low risk	Low risk	Low risk	Low risk	Low Risk of Bias, except for concerns about residual confounding
[85]	High risk	Some concerns	Some concerns	Some concerns	Low risk	Some concerns	Low risk	Some concerns
[33]	High risk	Some concerns	Some concerns	Low risk	Low risk	Some concerns	Some concerns	Some concerns
[114]	High risk	Some concerns	Some concerns	Low risk	Low risk	Some concerns	Some concerns	Some concerns
[80]	High risk	Some concerns	Some concerns	Some concerns	Some concerns	Some concerns	Some concerns	Some concerns
[86]	High risk	Some concerns	Some concerns	Low risk	Low risk	Some concerns	Some concerns	Some concerns
[87]	High risk	Some concerns	Some concerns	Some concerns	Some concerns	Some concerns	Some concerns	Some concerns
[119]	High risk	Some concerns	Some concerns	Some concerns	Low risk	Some concerns	Some concerns	Some concerns
[59]	High risk	High risk	Some concerns	Low risk	Low risk	Some concerns	Some concerns	High risk
[62]	High risk	Some concerns	Some concerns	Some concerns	Some concerns	Some concerns	Some concerns	Some concerns
[52]	High risk	Some concerns	Some concerns	Some concerns	Some concerns	Some concerns	Some concerns	Some concerns
[60]	High risk	Some concerns	Some concerns	Some concerns	Low risk	Some concerns	Some concerns	Some concerns
[51]	Some concerns	Some concerns	Some concerns	Some concerns	Low risk	Some concerns	Some concerns	Some concerns
[50]	High risk	Some concerns	Some concerns	Low risk	Some concerns	Some concerns	Some concerns	Some concerns
[58]	High risk	Some concerns	Some concerns	Low risk	Some concerns	Some concerns	Some concerns	Some concerns
[53]	High risk	Some concerns	Some concerns	Low risk	Some concerns	Some concerns	Some concerns	Some concerns

Study id.	Domain 1: Bias due to confounding	Domain 2: Bias arising from measurement of the exposure	Domain 3: Bias in selection of participants into the study (or analysis)	Domain 4: Bias due to post-exposure interventions	Domain 5: Bias due to missing data	Domain 6: Bias arising from measurement of the outcome	Domain 7: Bias in selection of the reported result	Global ROBINS-E
[54]	High risk	Some concerns	Some concerns	Some concerns	Some concerns	Some concerns	Some concerns	Some concerns
[55]	High risk	Some concerns	Some concerns	Some concerns	Some concerns	Some concerns	Some concerns	Some concerns
[56]	High risk	Some concerns	Some concerns	Low risk	Low risk	Some concerns	Some concerns	Some concerns
[57]	High risk	Some concerns	Some concerns	Low risk	Low risk	Some concerns	Some concerns	Some concerns
[41]	Some concerns	Some concerns	Some concerns	Some concerns	Low risk	Some concerns	Some concerns	Some concerns
[26]	High risk	Some concerns	Some concerns	Some concerns	High risk	Some concerns	Some concerns	Some concerns
[61]	High risk	Some concerns	Some concerns	Low risk	Low risk	Some concerns	Some concerns	Some concerns
[63]	High risk	Some concerns	Some concerns	Low risk	Some concerns	Some concerns	Some concerns	Some concerns
[40]	High risk	Some concerns	Some concerns	Some concerns	Some concerns	Some concerns	Some concerns	Some concerns
[39]	High risk	Some concerns	Some concerns	Some concerns	Some concerns	Some concerns	Some concerns	Some concerns
[38]	High risk	Some concerns	Some concerns	Low risk	Low risk	Some concerns	Some concerns	Some concerns
[27]	High risk	Some concerns	Some concerns	Low risk	Low risk	Some concerns	Some concerns	Some concerns
[29]	High risk	Some concerns	Some concerns	Low risk	Low risk	Some concerns	Some concerns	Some concerns
[31]	High risk	Some concerns	Some concerns	Some concerns	Low risk	Some concerns	Some concerns	Some concerns
[37]	Some concerns	Some concerns	Some concerns	Low risk	Some concerns	Some concerns	Some concerns	Some concerns
[34]	High risk	Some concerns	Some concerns	Low risk	Some concerns	Some concerns	Some concerns	Some concerns
[35]	High risk	Some concerns	Some concerns	Low risk	Some concerns	Some concerns	Some concerns	Some concerns
[30]	High risk	Some concerns	Some concerns	Some concerns	Low risk	Some concerns	Some concerns	Some concerns
[36]	Some concerns	Some concerns	Some concerns	Low risk	Low risk	Some concerns	Some concerns	Some concerns
[44]	High risk	Some concerns	Some concerns	Some concerns	Low risk	Some concerns	Some concerns	Some concerns
[45]	High risk	Some concerns	Some concerns	Some concerns	Non détaillé	Some concerns	Some concerns	Some concerns
[42]	High risk	Some concerns	Some concerns	Some concerns	Low risk	Some concerns	Low risk	Some concerns
[43]	High risk	Some concerns	Some concerns	Low risk	Low risk	Some concerns	Some concerns	Some concerns
[46]	High risk	Some concerns	Some concerns	Low risk	Some concerns	Some concerns	Some concerns	Some concerns
[145]	High risk	Some concerns	Some concerns	Some concerns	Some concerns	Some concerns	Some concerns	Some concerns

Study id.	Domain 1: Bias due to confounding	Domain 2: Bias arising from measurement of the exposure	Domain 3: Bias in selection of participants into the study (or analysis)	Domain 4: Bias due to post-exposure interventions	Domain 5: Bias due to missing data	Domain 6: Bias arising from measurement of the outcome	Domain 7: Bias in selection of the reported result	Global ROBINS-E
[113]	High risk	Some concerns	Some concerns	Low risk	Some concerns	Some concerns	Some concerns	Some concerns
[115]	Some concerns	Some concerns	Some concerns	Low risk	Some concerns	Some concerns	Some concerns	Some concerns
[116]	High risk	Some concerns	Some concerns	Low risk	Some concerns	Some concerns	Some concerns	Some concerns
[117]	High risk	Some concerns	Some concerns	Low risk	Low risk	Some concerns	Some concerns	Some concerns
[123]	High risk	Some concerns	Some concerns	Low risk	Some concerns	Some concerns	Some concerns	Some concerns
[124]	High risk	Some concerns	Some concerns	Low risk	Low risk	Some concerns	Some concerns	Some concerns
[126]	High risk	Some concerns	Some concerns	Low risk	Low risk	Some concerns	Some concerns	Some concerns
[127]	Some concerns	Some concerns	Some concerns	Low risk	Some concerns	Some concerns	Some concerns	Some concerns
[92]	High risk	Some concerns	Some concerns	Some concerns	Low risk	Some concerns	Some concerns	Some concerns
[98]	High risk	Some concerns	Some concerns	Some concerns	Low risk	Some concerns	Some concerns	Some concerns
[99]	High risk	Some concerns	Some concerns	Low risk	Low risk	Some concerns	Some concerns	Some concerns
[97]	Some concerns	Some concerns	Some concerns	Low risk	Some concerns	Some concerns	Some concerns	Some concerns
[93]	Some concerns	Some concerns	Some concerns	Low risk	Low risk	Some concerns	Some concerns	Some concerns
[94]	Some concerns	Some concerns	Some concerns	Low risk	Some concerns	Some concerns	Some concerns	Some concerns
[95]	High risk	Some concerns	Some concerns	Low risk	Low risk	Some concerns	Some concerns	Some concerns
[96]	High risk	Some concerns	Some concerns	Low risk	Low risk	Some concerns	Some concerns	Some concerns
[90]	High risk	Some concerns	Some concerns	Low risk	Some concerns	Some concerns	Some concerns	Some concerns
[91]	Some concerns	Some concerns	Some concerns	Low risk	Low risk	Some concerns	Some concerns	Some concerns
[89]	High risk	Some concerns	Some concerns	Some concerns	Low risk	Some concerns	Some concerns	Some concerns
[101]	High risk	Some concerns	Some concerns	Some concerns	Some concerns	Some concerns	Low Risk	Some concerns
[102]	High risk	Some concerns	Some concerns	Low risk	Some concerns	Some concerns	Some concerns	Some concerns
[103]	Some concerns	Some concerns	Some concerns	Low risk	Some concerns	Some concerns	Some concerns	Some concerns
[104]	High risk	Some concerns	Some concerns	Some concerns	High risk	Some concerns	Some concerns	High risk
